# Cellular and molecular effects of hyperglycemia on ion channels in vascular smooth muscle

**DOI:** 10.1007/s00018-020-03582-z

**Published:** 2020-06-27

**Authors:** Madeline Nieves-Cintrón, Víctor A. Flores-Tamez, Thanhmai Le, Miguel Martín-Aragón Baudel, Manuel F. Navedo

**Affiliations:** Department of Pharmacology, University of California Davis, One Shields Avenue, Davis, CA 95616 USA

**Keywords:** Myogenic tone, Vasculopathies, Diabetes

## Abstract

Diabetes affects millions of people worldwide. This devastating disease dramatically increases the risk of developing cardiovascular disorders. A hallmark metabolic abnormality in diabetes is hyperglycemia, which contributes to the pathogenesis of cardiovascular complications. These cardiovascular complications are, at least in part, related to hyperglycemia-induced molecular and cellular changes in the cells making up blood vessels. Whereas the mechanisms mediating endothelial dysfunction during hyperglycemia have been extensively examined, much less is known about how hyperglycemia impacts vascular smooth muscle function. Vascular smooth muscle function is exquisitely regulated by many ion channels, including several members of the potassium (K^+^) channel superfamily and voltage-gated L-type Ca^2+^ channels. Modulation of vascular smooth muscle ion channels function by hyperglycemia is emerging as a key contributor to vascular dysfunction in diabetes. In this review, we summarize the current understanding of how diabetic hyperglycemia modulates the activity of these ion channels in vascular smooth muscle. We examine underlying mechanisms, general properties, and physiological relevance in the context of myogenic tone and vascular reactivity.

## Introduction

According to recent reports by the World Health Organization and the American Diabetes Association, diabetes is a prevalent health problem reaching epidemic proportions [1, 2]. Diabetes is a multifactorial metabolic disorder characterized by the inability of the body to produce, secrete, and/or use the hormone insulin, which regulates glucose homeostasis [3–5]. As a consequence of this insulin dysfunction, the concentration of extracellular glucose is significantly elevated, poorly controlled or both. Elevated extracellular glucose level or hyperglycemia, is a hallmark metabolic abnormality in diabetes and a major risk factor for cardiovascular diseases, including heart attacks, hypertension, and stroke [3, 5–8]. Hyperglycemia is also associated with decreased cognitive function, retinopathy and nephropathy [9]. These pathological conditions have in common a link between hyperglycemia and alterations in small-resistance artery function [10, 11].

Small-resistance arteries and arterioles (< 150 μm in diameter) are composed of connective tissue, endothelial cells lining the vessel lumen, and smooth muscle cells wrapping around the vessel. These blood vessels are critical for nutrient delivery as well as for control of blood flow, tissue perfusion, and total peripheral resistance. Small-resistance arteries respond to increases in intravascular pressure by constricting, a process known as the myogenic or vascular tone [12]. Myogenic tone is influenced by the sympathetic system and neurohumoral vasoactive signals that induce constriction or dilation to maintain the moment-to-moment control of blood flow and tissue perfusion, which is critical for organ function [13]. The myogenic tone is an intrinsic property of vascular smooth muscle cells [14–16]. These cells receive and integrate many inputs, including variations in intraluminal pressure, as well as vasoconstrictor and vasodilatory queues from endothelial cells and nerve terminals innervating the vessels, to regulate their function [14]. These inputs help fine-tune vascular smooth muscle excitability and contractile state, and thus have major influence over arterial diameter, myogenic tone and vascular reactivity.

Vascular smooth muscle excitability and contractile state is modulated by Rho-associated kinase (ROK) dependent mechanisms [17, 18], and the activity of several ion channels [14, 19]. Agonists- and pressure-mediated activation of ROK are involved in Ca^2+^ sensitization of vascular smooth muscle contraction via inhibition of myosin light chain phosphatase and induction of actin polymerization [17, 18, 20]. In addition, the integrated activity of various ion channels expressed in vascular smooth muscle facilitates the control of membrane potential (V_M_) and the magnitude of intracellular Ca^2+^ concentration ([Ca^2+^]_i_) [14, 19]. Voltage-gated (K_V_), Ca^2+^-activated (BK_Ca_) and ATP-sensitive (K_ATP_) K^+^ channels, ryanodine receptors (RyR), IP_3_ receptors (IP_3_R), and voltage-gated Ca^2+^ channels are critical for regulation of V_M_ and [Ca^2+^]_i_ [14]. Thus, changes in the functional expression of these channels may alter vascular smooth muscle function and significantly impact the regulation of myogenic tone and vascular reactivity during metabolic challenges and pathological states, such as hyperglycemia and diabetes.

Impaired endothelial function is a well-recognized early modification contributing to altered myogenic tone during diabetic hyperglycemia (see recent comprehensive reviews [3, 7, 21–24]), although the role of endothelial ion channels is less clear. Thus, we thought it important to provide an overview of our current understanding of how ion channels in endothelial cells are impacted by hyperglycemia. There is also increasing appreciation that alterations in vascular smooth muscle function may contribute to diabetic vasculopathies. Particularly, changes in the functional expression of ion channels in vascular smooth muscle during diabetic hyperglycemia, the mechanisms underlying those changes, and their functional implications have received much attention in recent years [3, 14, 25–35]. The main goal of this review is to examine preceding and emerging data on cellular and molecular mechanisms by which hyperglycemia alters the function of vascular smooth muscle ion channels. This is important because ion channels are (1) major regulators of vascular smooth muscle function, myogenic tone and vascular reactivity, (2) key targets for many drugs, and (3) the mechanisms by which they are modulated by hyperglycemia may reveal new opportunities for therapeutic intervention. We will focus our discussion on K_V_/BK_Ca_/K_ATP_ K^+^ channels, RyR, IP_3_R and voltage-gated L-type Ca_V_1.2 channels as the impact of hyperglycemia on their expression and function has been well characterized. We will describe general properties of these ion channels, underlying mechanisms by which hyperglycemia and diabetes alter their function and the patho-physiological relevance in the context of myogenic tone and vascular reactivity.

## Myogenic tone and diabetic hyperglycemia

Accumulating evidence over the last 30 years has clearly established abnormal regulation of basal myogenic tone in both diabetic patients and different animal models of diabetes [3, 14, 18, 25–29, 32, 35–38]. Note that several animal models of diabetes have been developed to better understand diabetic complications, including vasculopathies [39, 40]. The advantages and drawbacks of the different models have been extensively described elsewhere [39, 40]. A comprehensive and integrative understanding of mechanisms underlying abnormal myogenic tone during diabetes has been challenging to reconcile. This is because studies have described either no change [35, 36, 41, 42], reduced [29, 43–45], or enhanced [31, 32, 37, 46–50] tone in both diabetic patients and animal models of diabetes (see Table 1 for details).

**Table 1:** Summary of effects of diabetic hyperglycemia on myogenic tone

Reference	Species	Diabetic model	Vascular bed	Effect on tone	Mechanism
Bagi et al. [41]	Mouse	dB/dB	Coronary arteriole	↔	No change in basal tone, but impaired flow and agonist-induced vasodilation
McVeigh et al. [35]	Human	Type-2 diabetic	Forearm arteries	↔	No apparent change in basal flow, but impaired endothelium dependent and independent vasodilation
Miura et al. [36]	Human	Type-2 diabetic	Coronary arterioles	↔	No change in basal tone, but impaired vasodilation in response to K_ATP_ agonists and hypoxia
Nieves-Cintron et al. [42]	Human	Type-2 diabetic	Adipose arteries	↔	No change in basal tone, but likely impaired vasodilation
Ito et al. [43]	Rat	BBZDR/Wor	Ophthalmic artery	↓	Unknown endothelium-mediated vasodilatory signal
Kold-Petersen et al. [44]	Rat	Goto-Kakizaki	Coronary septal and cerebral arteries	↓	Decreased smooth muscle Ca^2+^ sensitivity
Abd-Elrahman et al. [45]	Rat	Goto-Kakizaki	Endothelium-denuded cerebral arteries	↓	Decreased smooth muscle Ca^2+^ sensitivity due to ↓ focal adhesion kinase (FAK) autophosphorylation downstream of integrins
Schofield et al. [29]	Human	Type 2 diabetic	Gluteal fat arteries	↓	Unknown
Bagi et al. [46]	Mouse	dB/dB	Skeletal muscle arteriole	↑	Enhanced production and release of endothelium-dependent, COX-derived vasoconstrictor prostaglandins
Nystoriak et al. [31]	Mouse	HFD	Cerebral arteries	↑	Increased Ca_V_1.2 activity due to Ca_V_1.2 S1928 phosphorylation in vascular smooth muscle
Syed et al. [32]	Mouse	HFD and STZ	Cerebral arteries	↑	AC5-mediated increased Ca_V_1.2 activity in vascular smooth muscle
Jarajapu et al. [47]	Rat	BBZDR/Wor	Cerebral arteries	↑	Age-dependent changes in tone of unknown origin
Ungvari et al. [48]	Rat	STZ	Skeletal arteriole	↑	Increased PKC signaling and L-type Ca^2+^ channel activity in arteriole smooth muscle
Sauve et al. [49]	Human	Type 2 diabetic	Skeletal muscle arteriole	↑	Unknown
Sauve et al. [49]	Mouse	HFD/STZ combination	Olfactory and mesenteric arteries	↑	TNF-mediated enhancement of sphingosine-1 phosphatase signaling
Zimmerman et al. [50]	Rat	STZ	Cerebral arteries	↑	Decreased endothelium-mediated tonic NO release that impairs vascular smooth muscle K_ATP_ response
Velmurugan et al. [37]	Mouse	dB/dB	Mesenteric arteriole	↑	Decreased Nrf2 expression leading to increased ROS production
Lagaud et al. [54]	Mouse	dB/dB	Mesenteric arteries	↔ young ↑ older	In older mice, enhanced PKC activity and release of endothelium-dependent, COX-derived vasoconstrictor prostaglandins

In studies where myogenic tone was not different between non-diabetic and diabetic arteries, impaired arterial function in response to a given vasodilatory stimulus was still observed [35, 36, 41, 42]. In studies in which reduced basal myogenic tone during diabetes was observed, changes were correlated with decreased Ca^2+^ sensitization of the contractile machinery in smooth muscle [29, 43–45]. Accordingly, using cerebral vascular smooth muscle from Goto–Kakizaki (type-2 diabetes model) rats, reduced basal myogenic tone during diabetes was correlated with a decrease in focal adhesion kinase (FAK) autophosphorylation downstream of integrins [20, 45]. This breakdown in pressure-mediated FAK autophosphorylation appears to impair ROK signaling involved in myosin light chain phosphatase inhibition and actin polymerization [20, 45]. Intriguingly, a recent independent study using human internal mammary arteries from non-diabetic and diabetic patients found increased expression of contractile markers, as well as enhanced phosphorylation of proteins associated with ROK signaling activation and actin polymerization [51]. Although this study did not evaluate myogenic tone, one may speculate that human internal mammary arteries from diabetic patients may be either more constricted or hyperreactive to contractile agonists. Consistent with this possibility, a number of studies have shown ROK-mediated hyperreactivity of vascular smooth muscle cells in response to contractile stimuli in animal models of diabetes [48, 52, 53].

Conversely, studies showing increased myogenic tone in diabetes have described a number of mechanisms that include changes in functional expression of ion channels and signaling molecules controlling smooth muscle contractile state [31, 32, 37, 46–50]. The description of various mechanisms could result from the use of different species, animal models of diabetes, vascular beds and the severity of diabetes and hyperglycemic state. Intriguingly, a longitudinal study found distinct myogenic tone impairment with age during diabetes [54]. The study described no changes in myogenic tone in mesenteric arteries from dB/dB (type-2 diabetes model) mice compared to wild type control mice at 8 weeks of age [54]. However, myogenic tone was significantly elevated at 12 and 16 weeks of age [54]. These results were recapitulated in an independent study, which also demonstrated that the enhanced myogenic constriction in dB/dB mesenteric arteries was independent of endothelial influence [37], thus directly suggesting impairment of vascular smooth muscle function in diabetes. Moreover, although sex differences are well apparent in the context of diabetes [55, 56], how myogenic tone can be influenced by biological sex during this pathological condition is understudied. A recent study using STZ-treated Sprague Dawley rats found a significant change in the NO and endothelium-dependent hyperpolarizing factor (EDHF) signaling response in female mesenteric arteries compared to males [57], but no mechanisms or examination of effects on myogenic tone were provided. Together with the studies described above, these results suggest distinct myogenic/contractile responses during diabetes that may be influenced by age, biological sex, species, animal model of diabetes, vascular beds and degree of pathological state. These may be key confounding factors that should be taken into consideration when evaluating myogenic response during diabetes. Certainly, parallels and key information can be extracted from all these studies, which may be useful in identifying novel targets that could be exploited for improving the treatment of diabetic vasculopathy.

Similar to diabetes, the effects of acute and/or short-term hyperglycemia on myogenic tone are highly dependent on species, vascular bed, and the concentration of external glucose used (see Table 2 for details). Short-term glucose effects in vascular smooth muscle could also be influenced by sex, yet most studies have been performed using male tissue, thus highlighting the need to consider this biological variable in future experiments. Nonetheless, in posterior cerebral arteries from Wistar rats, equilibration of pressurized arteries in 44 mM d-glucose caused a reduction in myogenic tone over a range of pressures, compared to arteries equilibrated in 5 mM d-glucose [58]. The acute glucose effects on myogenic tone were prevented in endothelium-denuded arteries [58], thus indicating a role for an unknown endothelium-mediated vasodilatory signal. The mechanisms underlying the inhibition of myogenic tone during hyperglycemia in this model, however, are unknown. Conversely, in mesenteric arteries from Wistar rat, acute equilibration of pressurized arteries in 44 mM d-glucose did not change basal myogenic tone compared to control (5.5 mM d-glucose) [34]. However, myogenic tone in arteries exposed to 44 mM d-glucose was significantly elevated in response to activation of protein kinase C (PKC) or high K^+^ compared to controls [34]. These results suggest that elevated glucose potentiate responses that promote vascular smooth muscle contraction, such as PKC activation and membrane depolarization. In Sprague Dawley rat ophthalmic arteries, a glucose concentration-dependent effect was reported [43]. Accordingly, whereas 25 mM d-glucose significantly increased myogenic tone over a range of intraluminal pressures, 40 mM d-glucose had the opposite effect compared to control conditions (e.g. ~ 4 mM d-glucose) [43]. These differences were not observed in experiments using equimolar concentrations of mannitol and were abrogated in endothelial-denuded arteries [43]. These results suggest that endothelium-mediated signals drive glucose-induced differential changes in myogenic tone in rat ophthalmic arteries; the identity and mechanisms by which they act remain to be established. In C57BL6/J wild type mouse pressurized cerebral arteries, an acute elevation in external d-glucose from 10 to 20 mM caused robust constriction and increased tone [31, 32, 59]. Glucose-induced increased cerebral arterial tone in these studies was independent of endothelial function as a similar response was observed in endothelium-denuded arteries [31]. The mechanism involves activation of a novel signaling pathway resulting in regulation of ion channels and increased [Ca^2+^]_i_ in vascular smooth muscle (see extended description in sections below) [31, 32, 59]. Similarly, acute elevations in glucose from 4 to 14 mM d-glucose cause a robust constriction of rat parenchymal arterioles from cortical brain slices [60]. This glucose-induced constriction was linked to inhibition of K_V_ channels via a PKC-mediated mechanism [60]. These results suggest distinct regulation of myogenic tone in response to hyperglycemia in different vascular beds, animal models and hyperglycemic state. Given the importance of ion channels in both vascular endothelial and smooth muscle in controlling the myogenic response [14, 61], it is likely that their altered functional expression may contribute to changes in vascular response during diabetic hyperglycemia. In the next sections, we describe studies that have made direct links between changes in ion channels expression and function and changes in myogenic tone and vascular reactivity.

**Table 2:** Summary of effects of acute exposure to elevated glucose on myogenic tone

Reference	Species	[Glucose]	Vascular bed	Effect on tone	Mechanism
Cipolla et al. [58]	Rat	44	Cerebral arteries	↓	Unknown endothelium-mediated vasodilatory signal
Cipolla [34]	Rat	44	Mesenteric arteries	↔	No apparent change in basal flow, but ↑ PKC activity and sensitivity to depolarizing stimuli
Ito et al. [43]	Rat	25	Ophthalmic artery	↑	Unknown endothelium-mediated vasoconstricting signal
Ito et al. [43]	Rat	40	Ophthalmic artery	↓	Unknown endothelium-mediated vasodilatory signal
Nystoriak et al. [31] Syed et al. [32] Prada et al. [59]	Mouse	20	Cerebral arteries	↑	P2Y_11_/AC5-mediated increased Ca_V_1.2 activity due to Ca_V_1.2 S1928 phosphorylation
Straub et al. [60]	Rat	14	Cerebral arterioles	↑	K_V_ channel Inhibition via PKC-mediated pathway

## Endothelial ion channels and diabetic hyperglycemia

**Fig. 1:**
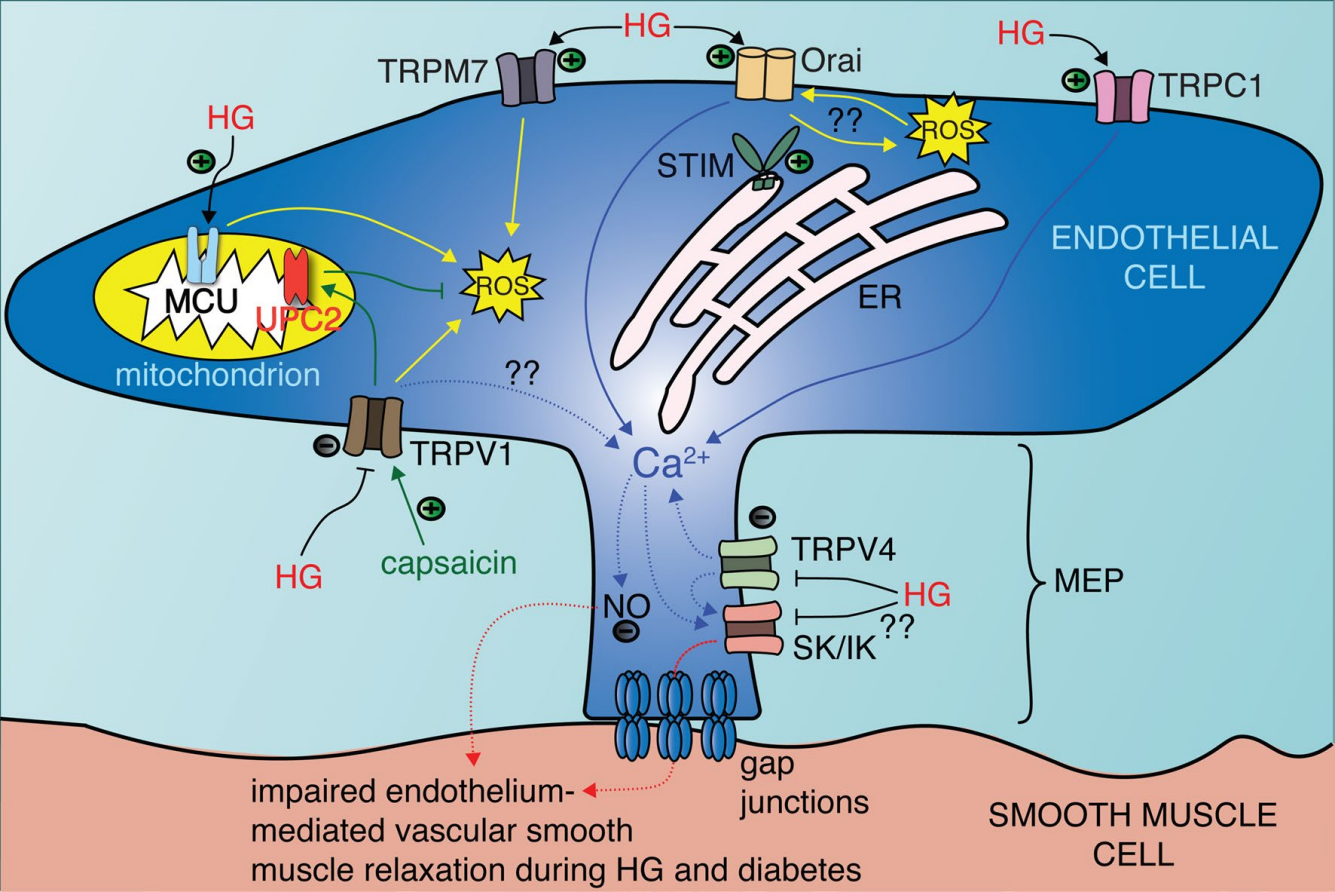
Schematic of mechanisms by which diabetic hyperglycemia (HG) alters endothelial ion channels and vascular smooth muscle excitability. Diabetic hyperglycemia distinctively modulates the activity of various ion channels in endothelial cells to alter ROS production, intracellular Ca^2+^ concentration and other signaling pathways that impair endothelium-dependent regulation of vascular smooth muscle cells. ?? denotes unknown mechanisms by which HG alters a particular target. Red dotted lines denotes reduced effects. *MEP* myoendothelial projections. Arrows = activation, lines with bars = inhibition

As cells lining the lumen of vessels, vascular endothelial cells are directly exposed to changes in blood glucose concentration. Glucose uptake in endothelial cells is mediated via the insulin-independent glucose transporter Glut1 [62, 63]. Therefore, high glucose levels in the external milieu may result in high glucose concentrations inside endothelial cells. This apparent lack of autoregulation could render endothelial cells more vulnerable to conditions, such as intermittent and chronic hyperglycemic states, which are known to alter endothelial function in different diabetic animal models and humans with diabetes [64–66]. In the context of myogenic tone regulation, endothelial dysfunction denotes the inability of the endothelium to promote vasodilation. Endothelial function is, at least in part, governed by a network of ion channels expressed in endothelial cells [61, 67]. These include (but not limited to) several types of Ca^2+^-activated K^+^ channels, a number of transient receptor potential (TRP) channels, store-operated channels and mitochondrial channels. These endothelial ion channels facilitate and/or promote the transmission of electrical signals and release of vasoactive signals to control vascular smooth muscle excitability and contractile state [61, 67]. Surprisingly, there is a limited understanding of how changes in endothelial ion channel functional expression contribute to endothelial dysfunction and impaired vasodilation during diabetic hyperglycemia. Only a handful of studies have addressed this issue, likely because of the difficulty in isolating native endothelial cells [68]. Figure 1 provides a summary of our current understanding of how diabetic hyperglycemia impacts endothelial ion channels.

### Endothelial TRP channels and diabetic hyperglycemia

Endothelial cells express several members of the TRP superfamily of cationic channels, including surface membrane TRPC (canonical), TRPV (vanilloid), and TRPM (melastatin) channels [69, 70]. Among them, TRPV4 channels have emerged as key regulators of endothelial cell function and modulators of myogenic tone and vascular reactivity due to their prominent Ca^2+^ permeability [61, 70, 71]. These channels localize to the myoendothelial projections (sections of close interaction between endothelial and smooth muscle cells) [72] where they can physically and functionally interact with small (K_Ca_2.X) and intermediate (K_Ca_3.X) conductance, Ca^2+^-activated K^+^ channels (SK and IK, respectively) in vessels from many species, including humans [71, 73, 74]. Activation of SK/IK channels by Ca^2+^ influx via TRPV4 channels is thought to induce endothelial cell membrane hyperpolarization and vasodilation [71]. It is suspected that this could be a generalized mechanism controlling vascular tone and vasodilation in many blood vessels [71, 74–77].

A recent study found that exposing primary culture bovine retinal endothelial cells to 25 mM external d-glucose for 72 h causes a significant reduction in the expression and function of TRPV4 channels compared to control conditions (e.g. 5 mM external d-glucose in the same cells) [78]. This change in glucose-induced downregulation of TRPV4 functional expression was not observed when cells were incubated with equimolar concentrations of mannitol, ruling out osmotic effects [78]. A reduction in TRPV4 expression was also described in diabetic rat retinal arterioles from a type-1 diabetic model (streptozotocin—STZ) [78], and rat primary cultured mesenteric endothelial cells [74]. A similar decrease in SK channel expression was also observed in mesenteric endothelial cells from STZ-treated rats [74]. This reduction in TRPV4 and SK channel expression may contribute to impair endothelium membrane hyperpolarization that is typically transmitted to the adjacent smooth muscle cells via gap junctions at the myoendothelial projections or alter the release of vasodilatory factors. Consistent with this, downregulation of both TRPV4 and SK channel was correlated with impaired acetylcholine-induced relaxation in mesenteric arteries from STZ mice [74]. Intriguingly, a recent study found reduced interaction (rather than decreased expression) between TRPV4 and SK channels in endothelial cells from hypertensive patients and animal models of hypertension that correlated with impaired vasodilation [79]. The impaired vasodilation was corrected in animals treated with JNc-440, a small molecule that enhanced the interaction between TRPV4 and SK channels in mesenteric endothelial cells [79]. Whether this structural change in TRPV4 and SK channel interaction also occurs in diabetic endothelial cells and if it could be rectified with the use of the JNc-440 to improve endothelial function during diabetes are exciting possibilities. Moreover, it will be important to define the mechanisms by which hyperglycemia alters TRPV4 and SK channel expression, distribution and interaction, and whether TRPV4 and SK channel function are actually altered in native endothelial cells leading to endothelial dysfunction.

TRPM7, TRPC1 and TRPV1 are also expressed in endothelial cells [69, 70], and their role in these cells has been studied in the context of diabetic hyperglycemia [80–82]. An upregulation in the expression of TRPM7 in response to high glucose (30 mM d-glucose) incubation for 72 h was described in human umbilical vein endothelial cells (HUVEC) [80]. Increased TRPM7 expression was correlated with enhanced reactive oxygen species (ROS) signaling and cytotoxicity [80]. However, the mechanisms underlying glucose-induced TRPM7 upregulation, their link to endothelial cell injury and how these changes may influence vascular tone are unknown. In bovine aortic endothelial cells, TRPC1, but not TRPC3, TRPC4 and TRPC6, expression was significantly upregulated when cells were cultured in 25 mM d-glucose for 72 h [81]. These changes were associated with sustained Ca^2+^ entry in endothelial cell. Yet, the physiological implications of this change and the mechanisms mediating glucose-induced TRPC1 upregulation have not been established. Conversely, culture of porcine ileal endothelial cells in 30 mM external d-glucose for 12 h resulted in a significant reduction in the expression of TRPV1 channels that was correlated with increased ROS production, reduced NO synthesis, and impaired acetylcholine-induced vasodilation [82]. Similar results were observed in dB/dB mice [82]. Intriguingly, all of these glucose-induced changes were prevented/ameliorated by capsaicin, a well-known TRPV1 agonist [82]. The capsaicin effects were mediated by increased functional expression of the uncoupling protein 2 (UPC2) [82], an antioxidant protein that regulates ROS production [83]. These results raise the possibility that the use of dietary capsaicin, via its action on endothelial TRPV1 channels, could be considered to improve endothelial function, including vasodilation, during diabetic hyperglycemia. In summary, diabetic hyperglycemia may have distinct effects on endothelial TRP channels that contribute to altering endothelial function.

### Endothelial MCU and diabetic hyperglycemia

The mitochondrial Ca^2+^ uniporter (MCU) located in the inner mitochondrial membrane is a key ion channel mediating Ca^2+^ uptake in this organelle [84]. MCU function can modulate [Ca^2+^]_i_ and redox signaling [84], which can have profound implications for myogenic tone and vascular reactivity regulation. A recent study described a significant augmentation in MCU expression in HUVEC cells upon increasing concentrations of external d-glucose from 5 to 30 mM for 72 h [85]. Glucose-induced MCU upregulation was correlated with increased cytosolic and mitochondrial Ca^2+^ as well as enhanced ROS and mitochondrial superoxide production [85]. These changes were prevented in the presence of an MCU inhibitor [85]. Thus, data suggest that changes in MCU function could contribute to alter endothelial Ca^2+^ and ROS signaling during diabetic hyperglycemia. Yet, further studies are necessary to convincingly establish functional implications of these hyperglycemia-induced changes in endothelial MCU function in the context of myogenic tone and vascular reactivity regulation.

### Endothelial store-operated channels and diabetic hyperglycemia

Store-operated Ca^2+^ entry (SOCE) is a mechanism leading to sustained Ca^2+^ entry due to activation of surface membrane receptors and subsequent reduction of Ca^2+^ concentration in the sarco/endoplasmic reticulum (S/ER) [86]. The underlying proteins for SOCE in many cells, including endothelial cells, have been identified as the S/ER Ca^2+^ concentration ([Ca^2+^]_S/ER_) sensor STIM and the surface membrane Ca^2+^ channel Orai [87, 88]. It is now recognized that a reduction in [Ca^2+^]_S/ER_ promotes the dynamic clustering of STIM to stimulate the activity of Orai channels leading to SOCE [87, 88]. Endothelial cells express both STIM and Orai channels [89]. Although the role of SOCE mediated by STIM and Orai in endothelial cells is not well established, both proteins have been associated with proliferation, migration and angiogenesis [89]. Additionally, a role for STIM and Orai in mechanisms underlying endothelial dysfunction and vascular complications is emerging [90]. In the context of hyperglycemia, a significant increase in SOCE has been reported in HUVEC cultured for 96 h in 30 mM d-glucose compared to control (e.g. 5 mM d-glucose) [91]. Similar results were reported in independent experiments using endothelial cells from different vascular beds and species exposed to 25 mM d-glucose for 72 h, as well as samples from type-2 diabetic patients and animal models of diabetes, specifically the Akita mouse [92]. The increase in SOCE by elevated glucose was shown to be mediated by ROS signaling [91], and/or by a distinct upregulation in the expression (not increase store-operated channel activation) of the different STIM and Orai isoforms expressed in endothelial cells [92]. However, the impact of STIM and Orai over-expression and increased SOCE on endothelial-dependent vasodilation is unclear. One possibility is that increased SOCE during diabetic hyperglycemia stimulates the activity of the Ca^2+^-dependent phosphatase calcineurin to regulate gene expression via activation of the transcription factor nuclear factor of activated T cells (NFAT) [92]. Consistent with this possibility, a recent study found elevated NFAT signaling in the Akita diabetic mice, and NFAT inhibition improved endothelial function in this animal model [93]. Thus, NFAT-dependent changes in gene expression could contribute to the alteration of endothelial function, including endothelium-dependent vasodilation. Future studies should further expand on these and other intriguing possibilities.

### Further considerations

Endothelial cells express many other ion channels that are critical for their functional regulation. Examples include IP_3_R that are involved in myoendothelial projection Ca^2+^ signals triggering vasodilation [94], inward rectifying K^+^ (K_ir_) channels that boost vasodilation upon activation [95, 96], and TRPA1 channels that contribute to Ca^2+^ signaling in the myoendothelial projections to promote vasodilation in response to ROS signaling [97]. How these channels are impacted by hyperglycemia is unclear. The accumulation of ROS producing advanced glycation end products (AGEs) is an additional element contributing to endothelial dysfunction during prolonged diabetic hyperglycemia [98, 99]. AGE is a biochemical modification triggered in response to elevated glucose levels that attaches to different structures and proteins to modify their function [100]. AGEs bind to different cell surface receptors, including the receptor for advanced glycation end products (RAGE) [100]. The AGE/RAGE complex can trigger activation of several pathways that modulate myriad cellular events including endocytosis, cell signaling and gene expression [100]. Intriguingly, recent findings suggest that acute AGEs exposure impairs Ca^2+^ signaling in endothelial cells [101]. Ca^2+^ signaling in endothelial cells are important for their function as well as myogenic tone and vascular reactivity regulation [61]. Thus, AGE-mediated alterations in endothelial Ca^2+^ signaling may play a fundamental role in the development of endothelial dysfunction during diabetic hyperglycemia. Yet, the underlying mechanisms by which AGEs impair Ca^2+^ signaling in endothelial cells are not well understood. Thus, these knowledge gaps could be further explored in future research. In summary, the functional expression of several endothelial ion channels is altered by diabetic hyperglycemia via multiple mechanisms, and this may contribute to endothelial dysfunction.

## Vascular smooth muscle ion channels and diabetic hyperglycemia

Vascular smooth muscle excitability and contractile state is governed by a sophisticated interplay between different ionic conductances [14, 19, 102]. Among them, K_V_, BK_Ca_ and K_ATP_ channels, RyR, IP_3_R and voltage-gated Ca^2+^ channels are critical for regulation of V_M_ and [Ca^2+^]_i_ [14]. Interest in the mechanisms by which glucose mediates changes in vascular smooth muscle function have heightened in recent years. This is because initial studies examining the effects of hyperglycemia described a significant increase in [Ca^2+^]_i_ in several vascular beds, including rat tail arteries [103], and mouse cerebral arteries [31, 32, 59]. In addition, accumulating epidemiological and functional studies also suggest vascular smooth muscle dysfunction during diabetic hyperglycemia [3, 14, 25–34]. Changes in ion channels functional expression are likely to contribute to alterations in vascular smooth muscle function during diabetic hyperglycemia. In the next sections, we will discuss how ion channels are modulated by diabetic hyperglycemia and the implications for vascular smooth muscle function.

### Effects of hyperglycemia on L-type Ca^2+^ channels

L-type Ca^2+^ channels are comprised of a pore-forming Ca_V_1.2 α_1C_ subunit and auxiliary β3 (predominant isoform in vascular smooth muscle) [104], α_2_δ and γ subunits that modulate channel function [105]. As stated above, Ca^2+^ influx via these channels plays a prominent role in vascular smooth muscle contraction and the level of the myogenic tone in resistance arteries [14, 19]. Therefore, changes in their expression and/or function could significantly influence vascular smooth muscle excitability.

In the context of diabetic hyperglycemia, although there are reports indicating diminished or unaltered L-type Ca^2+^ channel activity [106–110], the majority of the studies report a significant increase in channel activity in different experimental diabetic models and stages of hyperglycemia [31, 32, 48, 59, 111–115]. These differences are likely a reflection of the use of different vascular beds, cultured versus freshly isolated cells, and presumably the magnitude and duration of the hyperglycemic state. Moreover, the discrepancies in L-type Ca^2+^ channel activity could be associated to the specific animal model and concomitant metabolic abnormalities, such as hypercholesterolemia [116, 117] and hyperinsulinemia, [118] which could affect channel function. Table 3 summarizes the effects of diabetic hyperglycemia on vascular smooth muscle L-type Ca^2+^ channels.

**Table 3:** Summary of effects of diabetic hyperglycemia on L-type Ca^2+^ channel activity

Reference	Species	Condition	Vascular bed	Effect on channel activity	Mechanism
Fulton et al. [106]	Rat	STZ	Aorta	↓	Unknown
Wang et al. [107]	Rat	STZ	Tail arteries	↓	Unknown
Wang et al. [107]	Rat	25 mM d-glu	Tail arteries	↓	Unknown
Carmines et al. [108]	Rat	STZ	Kidney afferent arterioles	↓	Unknown
Abebe et al. [109]	Rat	STZ	Aorta and mesenteric arteries	↔	–
Mulhern et al. [109]	Rat	STZ	Aorta	↔	–
White et al. [111]	Rat	STZ	Mesenteric arteries	↑	Unknown
Pinho et al. [113]	Mouse	STZ	Aorta	↑	Upregulation of PI3K
Wilde et al. [114]	Rat	HFD	Cerebral arteries	↑	Increased plasma fatty acids
Ungvari et al. [48]	Rat	STZ	Skeletal arteries	↑	PKC-dependent pathway
Ma et al. [115]	Rat	Goto-Kakizaki	Cerebral arteries	↑	Unknown
Ma et al. [115]	WKY rat	20 mM d-glu	Cultured cerebral arteries	↑	Unknown
Navedo et al. [112] Nystoriak et al. [31] Syed et al. [32] Prada et al. [59]	Mouse	20 mM d-glu, HFD and STZ	Cerebral arteries	↑	P2Y_11_/AC5-mediated Ca_V_1.2 S1928 phosphorylation

A recent series of studies systematically and comprehensively explored the immediate effects of acute elevations in extracellular glucose on vascular L-type Ca^2+^ channel activity [31, 32, 59, 112, 115]. These studies have consistently shown that application of elevated glucose (20 mM d-glucose) caused a robust increase in the whole-cell L-type Ca^2+^ current density and in the frequency of localized persistent Ca^2+^ influx events via L-type Ca^2+^ channels (persistent Ca_V_1.2 sparklets [119]) in freshly dissociated human adipose, rat and mouse cerebral, and mouse mesenteric and femoral vascular smooth muscle [31, 32, 59, 112, 115]. The increase in the frequency of persistent Ca_V_1.2 sparklets is important because this gating modality can significantly amplify Ca^2+^ influx and therefore promote vascular smooth muscle contraction [120, 121].

**Fig. 2:**
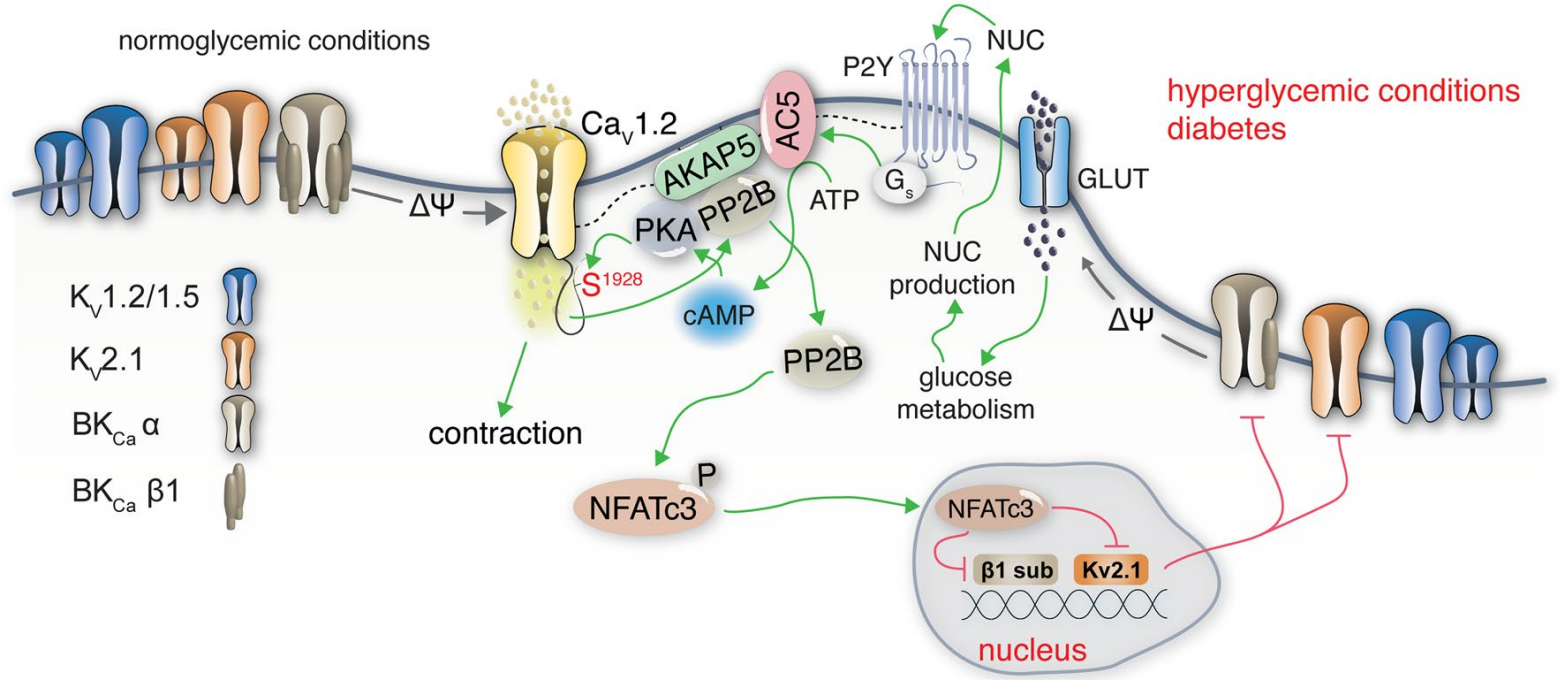
Schematic of a model for regulation of L-type Ca^2+^ channels and K^+^ channels during hyperglycemia and diabetes. In this model, the extent of Ca^2+^ influx via L-type Ca^2+^ channels play a key role in regulating excitation–contraction and excitation-transcription coupling. Under normoglycemic conditions, K^+^ channels, including K_V_1.X, K_V_2.X and BK_Ca_ channels, provide negative feedback control of pressure-induced membrane depolarization to constrain the activity of L-type Ca^2+^ channels, reduce global [Ca^2+^]_i_ and limit vascular smooth muscle contraction (see Ref. [19] for original work describing this concept). L-type Ca^2+^ channels are closely associated with signaling proteins, such as PKA, PP2B and AC5, through their scaffold by AKAP5. During hyperglycemic conditions, glucose is transported into the cells via a glucose transporter (GLUT). Inside the cell, metabolized glucose results in the production of nucleotides (NUC), such as ATP and UTP. These NUC are released to the extracellular space where they activate purinergic receptors coupled to G_s_ proteins (i.e. P2Y_11_). Activation of P2Y_11_ promotes AC5 activity and localized cAMP production. This cAMP microdomain can enable a pool of PKA that is intimately associated with L-type Ca^2+^ channels to increase Ca_V_1.2 phosphorylation at S1928, which will potentiate channel activity. Hyperactive L-type Ca^2+^ channels result in increased global [Ca^2+^]_i_ and contraction of vascular smooth muscle. The global increase in [Ca^2+^]_i_ also activates AKAP5-anchored PP2B resulting in dephosphorylation of the transcription factor NFATc3, which allows its nuclear translocation. Once in the nucleus, NFATc3 can regulate the expression of many genes, including K^+^ channels. Data have shown that glucose-induced NFATc3 activation leads to the selective suppression K_V_2.1 and BK_Ca_ β1 subunits. The reduction in the expression of these subunits decreases K_V_ currents and BK_Ca_ channel function, thus impairing the negative feedback membrane potential hyperpolarization. Ultimately these changes cause membrane potential depolarization, further Ca^2+^ influx via L-type Ca^2+^ channels and vascular smooth muscle contraction, thus creating a feedforward mechanism that could perpetuate the system under chronic hyperglycemic conditions. Green lines represent activation pathways and red lines represent inhibition pathways. Δ*ψ* = change in membrane potential. Model based on work from Refs. [31, 32, 59, 112, 165, 200, 210].

In one study, glucose-induced potentiation of L-type Ca^2+^ channel activity was prevented in WKY rat cerebral smooth muscle incubated in the presence of Salidroside, an active ingredient in *Rhodiola rosea* [115]. This compound has broad bioactive effects that can regulate inflammation, apoptosis, and the production of ROS signaling [122]. Considering that ROS is increased by elevated glucose [123], and ROS signaling can modulate L-type Ca^2+^ channel activity [124–126], it is tempting to speculate that glucose-induced ROS contributes to potentiation of L-type Ca^2+^ channels and that salidroside prevents this change in vascular smooth muscle by inhibiting ROS production. In additional independent studies, elevated glucose effects on the potentiation of L-type Ca^2+^ channel activity were found to require glucose transport into the cell and metabolization [31, 59, 112]. Indeed, application of the nonpermeable mannitol or non-metabolizable L-glucose had no effect on channel activity (Fig. 2). Moreover, vascular smooth muscle cells pre-incubated with indinavir, a broad glucose transporter inhibitor, ablated the elevated glucose-induced potentiation of L-type Ca^2+^ channel activity [31], which is consistent with the idea that facilitative glucose transport may contribute to modulating vascular reactivity [127]. Using cerebral arteries as a model system, glucose-induced potentiation of L-type Ca^2+^ channels was correlated with enhanced myogenic tone through an endothelium-independent mechanism [31, 32, 59]. Similar acute glucose-induced changes in myogenic tone have been observed in mesenteric arteries [128]. These results highlight the physiological relevance of the glucose effects on vascular smooth muscle L-type Ca^2+^ channels. Moreover, results raise a need to understand the mechanisms by which glucose regulate L-type Ca^2+^ channel activity in vascular smooth muscle and perhaps other excitable and non-excitable cells.

### How does glucose stimulate vascular L-type Ca^2+^ channel activity?

Increased PKC activity in vascular smooth muscle has been extensively reported during diabetic hyperglycemia [129–132]. PKC activity is also known to be required for persistent vascular L-type Ca^2+^ channel events as well as for increased channel activity during other pathological conditions, such as hypertension [119, 133–137]. Thus, it was reasonable to hypothesize that enhanced PKC activity during an elevated glucose challenge would underlie enhanced L-type Ca^2+^ channel activity. Yet, when using vascular smooth muscle cells from PKC knockout mice, it was found that glucose was still able to stimulate L-type Ca^2+^ channel activity [112]. Rather, glucose-induced potentiation of L-type Ca^2+^ channels was prevented in wild type and PKC knockout cells that were pre-treated with a protein kinase A (PKA) inhibitor [112]. Moreover, glucose-induced constriction was also prevented in pressurized arteries exposed to a PKA inhibitor [31]. These results suggest that glucose-induced vascular L-type Ca^2+^ channel potentiation leading to vasoconstriction is mediated by PKA activity (Fig. 2) [31, 112]. These observations were unexpected as a role for PKA in modulating vascular L-type Ca^2+^ channels has not been previously established [138]. Moreover, PKA activity has been usually linked to relaxation of vascular smooth muscle, and therefore vasodilation [139–143]. The observation that glucose mediates vascular L-type Ca^2+^ channel potentiation, vascular smooth muscle contraction and vasoconstriction via a PKA-dependent pathway could be reconciled if elevated glucose is predominantly stimulating a subpopulation of PKA molecules near L-type Ca^2+^ channels while avoiding the activation of other PKA pools associated with signaling pathways underlying vascular smooth muscle relaxation. In other words, hyperglycemia would mainly activate PKA molecules that are compartmentalized with L-type Ca^2+^ channels, thus facilitating their potentiation.

Compartmentalization of proteins like PKA can be facilitated by scaffold proteins, such as A-kinase anchoring proteins (AKAPs). In doing so, AKAPs fine-tune signal transduction in multiple cells by positioning signaling generators, regulatory enzymes, and effector proteins in close proximity to a specific substrate [144–146]. The AKAP5 isoform (murine AKAP150 and human AKAP79) interacts with adenylyl cyclase (AC), PKA, PKC and Ca_V_1.2 [147–152]. Therefore, this scaffold could mediate compartmentalization of PKA and the glucose-induced, PKA-mediated stimulation of vascular L-type Ca^2+^ channels. Consistent with this possibility, super-resolution localization maps generated with ground state depletion microscopy, and complementary proximity ligation assay confirmed close spatial association between PKA and Ca_V_1.2 in native human adipose and mouse cerebral vascular smooth muscle cells that were dependent on expression of AKAP5 [31]. Indeed, it was found that genetic depletion of AKAP5 increased the distance between pools of PKA and Ca_V_1.2 clusters and prevented the stimulation of L-type Ca^2+^ channels by glucose in mouse cerebral smooth muscle [31]. Moreover, the use of genetically modified mice expressing an AKAP5 that could not bind PKA or PKC confirmed an essential role for compartmentalized PKA, but not compartmentalized PKC, in mediating the glucose effects on L-type Ca^2+^ channel activity and cerebral artery reactivity [31]. Together with prior studies on the role of PKA in vascular smooth muscle relaxation [139–143], findings suggest unappreciated mechanisms by which PKA may differentially regulate the contractile state of these cells (Fig. 2).

AKAP5-anchored PKA regulation of L-type Ca^2+^ channel activity and vascular reactivity upon increased glucose was found to be mediated by direct phosphorylation of the pore-forming Ca_V_1.2 subunit at serine 1928 (S1928) (Fig. 2) [31]. This amino acid residue is a highly conserved PKA phosphorylation site [153], which has been linked with PKA-dependent regulation of L-type Ca^2+^ channels in neurons and cardiac cells [151, 154–157]. In cardiac myocytes, however, the functional relevance of S1928 phosphorylation is unclear [158–160], as PKA regulation of cardiac L-type Ca^2+^ channels was normal in cells from knockin mice with disrupted phosphorylation of S1928 (e.g. S1928A mice) [158] or 21 other putative PKA sites [159]. In stark contrast, S1928 plays a key role on AKAP5-anchored PKA-dependent regulation of vascular Ca_V_1.2 [31]. Accordingly, ablation of AKAP5 or disruption of the interaction between AKAP5 and PKA prevented glucose-induced increase in S1928 phosphorylation [31]. Elevated glucose failed to potentiate L-type Ca^2+^ channel activity and increase global [Ca^2+^]_i_ in vascular smooth muscle from S1928A mice [31]. In addition, no glucose-induced constriction was observed in S1928A cerebral arteries [31]. The complete loss of glucose effects on L-type Ca^2+^ channel activity and vascular reactivity just by the modification of a single Ca_V_1.2 amino acid was surprising since elevated glucose levels may also suppress the function of K^+^ channels (see below) [60, 128, 161, 162], which will depolarize vascular smooth muscle membrane potential leading to an increase in the open probability of L-type Ca^2+^ channels and an increase in global [Ca^2+^]_i_ [14, 163]. Using mathematical modeling that facilitates quantification of the relative role of many elements that interact nonlinearly to regulate arterial myocyte excitability [164], it was predicted that preventing the potentiation of L-type Ca^2+^ channels in a similar manner as that observed in S1928A experiments would prevent/ameliorate changes in global [Ca^2+^]_i_ despite concomitant modifications in K^+^ channel function and corresponding membrane depolarization [165]. Indeed, these predictions were confirmed experimentally [31, 165]. These results confirmed a predominant role for Ca_V_1.2 S1928 phosphorylation as a primary mechanism underlying [Ca^2+^]_i_ and vasoconstriction in response to elevated glucose. Altogether, these findings uncovered an important molecular link by which increased Ca_V_1.2 S1928 phosphorylation triggers the potentiation of vascular L-type Ca^2+^ channels leading to vasoconstriction upon glucose-induced AKAP5-anchored PKA activation. Whether Ca_V_1.2 S1928 phosphorylation may be relevant for vascular L-type Ca^2+^ channel regulation by other stimuli remains to be examined. Findings also highlight an unappreciated dichotomy in S1928 regulation between cardiac and vascular Ca_V_1.2 that suggest unique tissue-specific regulation. We speculate that this differential S1928 regulation of cardiac and vascular Ca_V_1.2 may be associated with the specific expression of different splice variants, distinct phosphorylation profiles, post-translational modifications of the Ca_V_1.2 subunit and interacting partners or unique interactome profile. Indeed, a recent report discovered that PKA-dependent Ca_V_1.2 regulation in the heart was mediated by Rad, a small Ras-like G protein, through an indirect interaction via the accessory Ca_V_ β subunit [166]. Whether Rad regulates Ca_V_1.2 in vascular smooth muscle and during diabetic hyperglycemia remains to be established.

### How does glucose activate PKA to stimulate vascular L-type Ca^2+^ channel activity?

The observation that elevated glucose stimulates vascular smooth muscle L-type Ca^2+^ channel activity via a PKA dependent pathway raised an important question: How does glucose activate PKA? The classic molecular machinery stimulating activity of an effector protein, such as PKA implicates upstream production of second messengers [e.g. cyclic adenosine monophosphate (cAMP)], as well as activation of regulatory enzymes (e.g. AC) and signaling generators [e.g. G protein-coupled receptors (GPCRs)] [144, 145]. Uncovering the identities of proteins that are activated in response to elevated glucose is important as it may identify novel signaling pathways and additional mechanisms for signaling compartmentalization that could help explain the intriguing effects of glucose-induced PKA activity on vascular smooth muscle contractility.

Glucose has been shown to stimulate cAMP synthesis in yeast and murine pancreatic β cells [167, 168], thus raising the possibility that a similar glucose-induced cAMP production could be observed in vascular smooth muscle. cAMP signaling has been extensively examined in vascular smooth muscle using conventional biochemical approaches [169]. However, these approaches do not provide spatiotemporal information about cAMP signaling that is critical for cellular regulation [170], including those in vascular smooth muscle [169]. To overcome this issue, investigators are beginning to express different FRET biosensors capable of measuring cAMP nanodomains in cultured vascular smooth muscle [32, 59, 171, 172]. One such FRET biosensors is the Epac1-camps-based FRET sensor typically known as ICUE3 [173]. The ICUE3 sensor can be targeted to different organelles and subcellular compartments [170], thus facilitating examination of cAMP signaling spatiotemporal dynamics in response to physiological and pathological stimuli, such as elevated glucose. Accordingly, using a membrane-targeted ICUE3 (but not nucleus-targeted ICUE3) biosensor expressed in unpassaged mouse aortic vascular smooth muscle cells, it was found that elevated glucose was able to induce a subtle, yet significant increase in cAMP synthesis that was further amplified by the broad AC activator forskolin [32, 59]. These results suggested localized cAMP synthesis upon elevated glucose, which may be essential for activation of a PKA pool that could trigger contraction rather than relaxation (Fig. 2). Consistent with this view, glucose caused contraction of vascular smooth muscle and vasoconstriction, while forskolin, which will engage all PKA pools, led to relaxation of vascular smooth muscle and vasodilation [31, 32].

The production of cAMP is mediated by AC isoforms. Nine membrane-bound AC isoforms have been identified [174]. In vascular smooth muscle, AC3, AC5 and AC6 are abundantly expressed [172, 175, 176]. AC6 and, to an extent, AC3 have been linked with β adrenergic regulation of the activity of various K^+^ channels that contributes to relaxation of vascular smooth muscle [172, 175]. Unsurprisingly, alterations in AC signaling has been reported in vascular smooth muscle upon elevated glucose and diabetes, which may contribute to cell proliferation, increased oxidative stress, impaired relaxation, and enhanced expression of contractile genes [51, 177–179]. Accordingly, basal AC activity was reduced in cultured A10 cell lines and rat aorta exposed to 26 mM d-glucose for 3–4 days, compared to control (5.5 d-glucose) [177]. The glucose-induced reduction in AC activity was subsequently associated with ROS production, particularly superoxide anion (O^2−^), as treatment with antioxidants prevented the changes in AC function [178]. In additional independent experiments, a reduction in AC6 expression was observed in rat mesenteric arteries [179]. The functional implications for this glucose-induced reduction in AC activity were mostly linked to changes in vascular smooth muscle proliferation and migration [129], although there is recent evidence of altered agonist-induced relaxation in isolated mesenteric artery rings [179]. Important for mechanisms underlying glucose activation of PKA, it was found that AC5 activity, but not AC6, was necessary for glucose-induced cAMP synthesis, L-type Ca^2+^ channel stimulation and vasoconstriction in mouse cerebral vascular smooth muscle/arteries (Fig. 2) [32]. Mathematical modeling and subsequent experimental validation confirmed that genetic ablation of AC5 normalized global [Ca^2+^]_i_ and vascular reactivity in response to elevated glucose [32]. These result suggest a detrimental role for AC5 in vascular smooth muscle upon elevated glucose. Intriguingly, results are similar to those observed in S1928A vascular smooth muscle cells [31, 165]. We thus speculate that in the absence of AC5 activity, cAMP/PKA-mediated phosphorylation of Ca_V_1.2 S1928 upon elevated glucose is unlikely to occur. It can be also speculated that AC5 and AC6/AC3 may have distinctive, yet critical roles in vascular smooth muscle function. Moreover, the specific activation of AC5 leading to localized AC5-dependent cAMP synthesis can provide two extra layers of compartmentalization that could facilitate PKA-dependent regulation of vascular smooth muscle excitability. Thus, results in this section highlight how changes in AC activity may modulate activation of different molecular pathways to alter vascular smooth muscle function in response to diabetic hyperglycemia.

AC signaling generation is triggered by the activation of G_s_ protein-coupled receptors (G_s_PCR) [145]. Within the vasculature, elevated glucose can stimulate autocrine release of nucleotides (e.g. ATP, UTP) into the extracellular space [59, 180, 181]. This can then activate P2Y receptors in vascular smooth muscle leading to increased [Ca^2+^]_i_ and changes in vascular smooth muscle contractile state [181–184]. While purinergic regulation of tone has been mostly associated with activation of G_q_/_i_-linked P2Y_2/4/6_ receptors [185], the P2Y_11_ is the only purinergic receptor coupled to G_s_, which can stimulate AC/PKA activity [186, 187]. P2Y_11_ receptors, however, have also been shown to hetero-oligomerize with P2Y_1_ and P2Y_6_ receptors and this structural arrangement can modulate P2Y_11_ activity and the underlying cellular response [188–192]. Thus, a role for P2Y_11_ in glucose-induced cAMP synthesis leading to PKA activation could be masked by the influence of other P2Y receptors.

In comprehensive experiments that address the aforementioned possibility, the expression for this purinergic receptor and its close proximity to PKA were confirmed in freshly dissociated human adipose vascular smooth muscle cells [59]. In these cells, the ICUE3 sensor revealed that elevated glucose and the selective P2Y_11_ agonist NF546 stimulated cAMP production to about the same magnitude, even when both stimuli were applied simultaneously [59]. The glucose/NF546-induced production of cAMP was blocked by the selective P2Y_11_ antagonist NF340 but not by selective inhibitors of P2Y_1_ and P2Y_6_ [59]. Moreover, results correlated with PKA-dependent enhancement of S1928 phosphorylation and L-type Ca^2+^ current potentiation by elevated glucose and NF546 [59]. These effects were blocked by the P2Y_11_, but not P2Y_1_ and P2Y_6_, antagonist [59]. These results are relevant because they suggest a key role for P2Y_11_ receptor function in mediating the elevated glucose effects in vascular smooth muscle that is independent of their potential hetero-oligomerization with P2Y_1_ or P2Y_6_ receptors. Comparable findings were observed using a robust approach in mouse cerebral artery vascular smooth muscle. Even though the P2Y_11_ gene has not been found at the expected location in the mouse genome, there has been recent rodent annotations for the P2Y_11_ gene (e.g. XM_008766009.2 and XM_0130655917.2) and a growing number of studies using pharmacological approaches that hint at the presence of at least a P2Y_11_-like receptor in mice [193]. Therefore, a P2Y_11_-like receptor may underlie the glucose response in murine vascular smooth muscle. Altogether, these findings indicate a role for P2Y_11_/P2Y_11_-like receptors as the signaling generators triggering the localized cAMP synthesis upon elevated glucose (Fig. 2). This glucose-induced local cAMP production can then activate a specific pool of PKA near Ca_V_1.2 to regulate L-type Ca^2+^ channel activity and vascular reactivity. Moreover, although a partnership between P2Y_11_ and AC5 remains to be established, we propose the assembly of a previously unrecognized macromolecular signaling complex formed by P2Y_11_, AC5, AKAP5-anchored PKA and Ca_V_1.2 (Fig. 2) [31, 32, 59, 112]. The formation and activation of this macromolecular complex could mediate glucose signaling in vascular smooth muscle and perhaps other excitable and non-excitable cells, which may have broad clinical and therapeutic implications.

### Effects of hyperglycemia on K^+^ channels

**Fig. 3:**
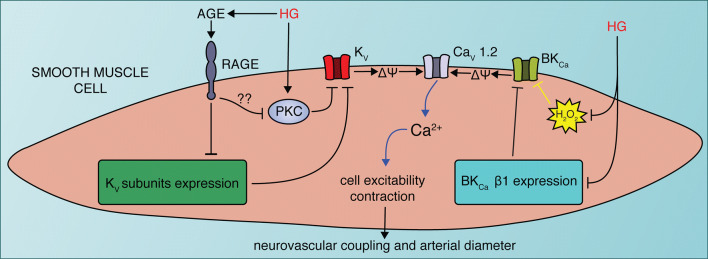
Schematic of mechanisms by which diabetic hyperglycemia (HG) alters vascular smooth muscle K^+^ channels. Multiple pathways have been described by which diabetic hyperglycemia can modulate the activity of K^+^ channels. Impaired K^+^ channels function has a significant impact on intracellular Ca^2+^ levels and contractile state of vascular smooth muscle, which will modulate arterial diameter and neurovascular coupling. Arrows = activation, lines with bars = inhibition, double question marks denotes unknown mechanisms by which HG alters a particular target, and Δ*ψ* = change in membrane potential

K^+^ channels regulate vascular smooth muscle membrane potential and therefore has a major impact on the control of [Ca^2+^]_i_, myogenic tone and vascular reactivity [14]. Activation of K^+^ channels relaxes vascular smooth muscle, whereas their inhibition leads to contraction [194]. Vascular smooth muscle cells express a number of isoforms from various types of K^+^ channels, including K_V_, BK_Ca_ and K_ATP_. In this section, we discuss the effects of hyperglycemia on the function of K_V_, BK_Ca_ and K_ATP_ channels and the physiological implications (Fig. 3). Table 4 provides a summary of different studies showing alterations in K^+^ channels activity and potential underlying mechanisms in response to diabetic hyperglycemia.

**Table 4:** Summary of effects of diabetic hyperglycemia on K^+^ channel activity

Reference	Species	Condition	Vascular bed	Effect on channel activity	Mechanism
Rainbow et al. [162] Jackson et al. [128]	Rat	Acute 10—20 mM d-glu	Mesenteric	↓ K_V_ activity	PKC-dependent pathway involving PKCα and PKCβ isoforms
Straub et al. [60]	Rat	Acute 14 mM D-glu	Cerebral arteriole	↓ K_V_ activity	PKC-dependent pathway
Liu et al. [161] Li et al. [201, 202]	Rat	23 mM D-glu for 24 h	Coronary arteriole	↓ K_V_ activity	Superoxide and peroxynitrite mediated inhibition of K_V_ channels
Bubolz et al. [234, 236]	Rat	STZ	Coronary arteriole	↓ K_V_ activity	↓ K_V_1.2 expression and ↑ K_V_1.2 nitration via peroxynitrite
Su et al. [199]	Rat	23 mM D-glu for 48 h and HFD	Coronary arteriole	↓ K_V_ activity	AGE/RAGE mediated reduction in functional expression of K_V_1.2/1.5
Nieves-Cintron et al. [200]	Mouse	20 mM D-glu for 48 h and HFD	Cerebral and mesenteric arteries	↓ K_V_ activity	Selective downregulation of K_V_2.1 subunit via AKAP5-anchored PP2B and NFAT
Morales-Cano et al. [204]	Rat	30 mM D-glu and STZ	Coronary arteries	↓ K_V_ activity	Impaired K_V_7 functional expression
Lu et al. [209]	Rat	22 mM D-glu for 72 h	Aorta and coronary arteries	↓ BK_Ca_ activity	ROS-mediated inhibition of the BK_Ca_ α subunit
Lu et al. [211, 245] Zhang et al. [241] Li et al. [247]	Rat and mouse	STZ, HFD	Aorta and coronary arteries	↓ BK_Ca_ activity	NFκB/MuRF1-mediated BK_Ca_ β1 degradation and Nrf2-mediated transcriptional regulation of the BK_Ca_ β1
Rueda et al. [242]	Mouse	dB/dB	Aorta	↓BK_Ca_ activity	Reduced BK_Ca_ β1/ BK_Ca_ α subunit ratio
Nystoriak et al. [210]	Mouse	20 mM D-glu for 48 h and HFD	Cerebral arteries	↓BK_Ca_ activity	Downregulation of BK_Ca_ β1 subunit via AKAP5-anchored PP2B and NFAT
Nieves-Cintron et al. [42]	Human	Type 2 diabetic patients	Adipose arteries	↓BK_Ca_ activity	Reduce functional coupling of BK_Ca_ α and β1 subunits
Lu et al. [243]	Human	Type 2 diabetic patients	Coronary arteries	↓BK_Ca_ activity	BK_Ca_ α and β1 subunits downregulation
Kawano et al. [214]	Rat	23 mM D-glu for 24 h	Aorta	↔ K_ATP_ activity	–
Zimmerman et al. [50] Kamata et al. [248] Bouchard et al. [249]	Rat	STZ	Aorta, cerebral and coronary arteries	↓K_ATP_ activity	Unknown
Miura et al. [36]	Human	Type-1 and type-2 diabetes	Coronary arteries	↓K_ATP_ activity	Unknown
Lee et al. [250]	Rat	STZ	Mesenteric arteries	↓K_ATP_ activity	Unknown, but activity is restored with KMUP-1
Li et al. [251]	Rat	STZ	Aorta	↓K_ATP_ activity	Oxidative stress-mediated inhibition due to K_ir_6.X S-glutathionation
Li et al. [252]	Human	Gestational diabetes	Umbilical arteries	↓K_ATP_ activity	Downregulation of K_ir_6.X and SUR2B subunits

### *K*_*V*_* channels*

Vascular smooth muscle cells express a wide variety of K_V_ subunits, including K_V_1 (K_V_1.1, K_V_1.2, K_V_1.3, K_V_1.5, K_V_1.6), K_V_2 (K_V_2.1), K_V_7 (K_V_ 7.1–5) and member of the silent K_V_ subunits (K_V_9.3) [14, 195–198]. It has long been recognized that the function of several of these K_V_ subunits can be distinctively modified by acute and short-term chronic hyperglycemia [14, 60, 128, 161, 162, 199–202]. Acute (5–10 min) elevations in glucose from 4–5 mM d-glucose to 15–20 mM d-glucose was found to inhibit K_V_ channel activity in vascular smooth muscle from rat mesenteric arteries and cerebral arterioles [60, 128, 162]. Acute glucose-mediated inhibition of K_V_ channels appears to involve PKC signaling [60, 162], specifically those associated with PKCα and PKCβ isoforms (Fig. 3) [128]. Glucose-induced K_V_ channel inhibition was subsequently correlated with vascular smooth muscle membrane depolarization and increased vasoconstriction (or myogenic tone) in rat mesenteric arteries (ex vivo) and cerebral arteriole (in situ) [60, 128, 162]. KCl^−^ and agonist-induced vasoconstriction were also elevated in rat mesenteric arteries [128]. Moreover, acute changes in extracellular glucose concentration were related to immediate in vivo alterations in neurovascular coupling [60], which is the modification of local perfusion due to changes in neuronal activity [203]. Glucose could modulate neurovascular coupling either by direct effects in the vasculature via mechanisms described above and/or by altering astrocytic function. However, how glucose stimulates PKC signaling, how PKC ultimately inhibits K_V_ channels and the mechanisms by which elevated glucose could impair neurovascular coupling are either unclear or unknown, thus opening new lines of research.

Chronic hyperglycemia (≥ 24 h incubation) has also been shown to cause a reduction in K_V_ channel activity in vascular smooth muscle [161, 199–202, 204]. Studies have found a decrease in K_V_ current density in rat coronary vascular smooth muscle incubated for 24 h in 23 mM d-glucose [161]. Glucose-induced reduction in K_V_ channel function was shown to be mediated by superoxide and peroxynitrite production with no apparent change in the expression of K_V_1.2 and K_V_1.5 subunits (other K_V_ subunits were not examined) [201, 202]. In independent experiments where rat coronary intact arteries rather than isolated vascular smooth muscle were incubated for 48 h in 23 mM d-glucose, a reduction in K_V_ function was also observed [199]. This change in K_V_ function was correlated with downregulation in the expression of K_V_1.2 and K_V_1.5 mRNA and protein levels [199]. The decreased functional expression of K_V_1.2/1.5 in response to elevated glucose was found to be mediated by the production of AGE and specifically the activation of RAGE, as inhibiting RAGE with an anti-RAGE antibody prevented both the reduction in K_V_1.2/1.5 protein levels and K_V_ current component (Fig. 3) [199]. How glucose-induced AGE/RAGE signaling leads to decreased functional expression of K_V_1.2/1.5-mediated K_V_ currents is currently unclear. Interestingly, AGE activity has been correlated with the stimulation of PKC in different diabetic settings [51, 205, 206]. Thus, it could be speculated that glucose-induced AGE signaling may trigger activation of PKC to regulate functional expression of K_V_ subunits during diabetic hyperglycemia. In additional experiments using vascular smooth muscle from rat coronary arteries incubated for 20 h in 30 mM d-glucose, the reduction in K_V_ channel function was also associated, at least in part, with a reduction in K_V_7 subunit functional expression [204]. The mechanisms for impaired K_V_7 function were correlated with downregulation of KCNQ1 and KCNQ2 expression [204]. Finally, in vascular smooth muscle from cerebral arteries incubated for 48 h in 20 mM d-glucose, a reduction in K_V_ current density was associated with selective downregulation of the K_V_2 subunit mRNA and protein levels via a mechanism that requires anchoring of the phosphatase calcineurin by AKAP5 (see further discussion below) (Fig. 2) [200]. These glucose-mediated changes in K_V_ function did not seem to alter resting vessel diameter, but rather impair vasoactive responses that could proceed via engagement of K_V_ channels in some studies [201, 202]. In others, the reduction in K_V_ function was correlated with increased vascular smooth muscle hypercontractility and enhanced myogenic tone [200, 204]. Altogether, results suggest that alterations in K_V_1.X, K_V_2.X and K_V_7.X subunit functional expression via various mechanisms during hyperglycemia may contribute to impair vascular smooth muscle function, myogenic tone and vascular reactivity. How acute and chronic hyperglycemia may affect other K_V_ subunits is unclear, thus opening opportunities for additional research.

### *BK*_*Ca*_* channels*

BK_Ca_ channels are abundantly expressed in vascular smooth muscle cells where they provide negative tonic feedback regulation of myogenic tone [14]. They are composed of a pore-forming α subunit and accessory β1 and γ subunits, which regulate Ca^2+^ sensitivity of the channel [14]. BK_Ca_ channels can be activated by membrane depolarization and localized increases in intracellular Ca^2+^ signals known as Ca^2+^ sparks [14, 105, 207]. This Ca^2+^ signals are produced by the opening of ryanodine receptors (RyR) located in the membrane of the sarcoplasmic reticulum [14, 105, 208]. Intriguingly, acute elevations in extracellular glucose do not seem to affect BK_Ca_ channel function in HEK cells expressing the pore-forming α subunit [209]. However, similar experiments examining the acute effects of elevated glucose in native vascular smooth muscle cells appear to be missing.

On the other hand, incubation of vascular smooth muscle in elevated glucose for as short as 48 h seems to induce a significant reduction in the activity of BK_Ca_ channels (see Table 4) [209, 210]. Accordingly, chronic incubation (72 h) of rat coronary vascular smooth muscle in 22 mM d-glucose resulted in a reduction in BK_Ca_ channel activity [209]. This glucose-induced inhibition of BK_Ca_ channel activity was reported to be mediated by increased oxidation of the BK_Ca_ α subunit at cysteine 911 by hydrogen peroxide (Fig. 3 and Table 4) [209]. In mouse cerebral vascular smooth muscle, a reduction in BK_Ca_ channel activity during chronic glucose incubation (20 mM d-glucose for 48 h) stemmed from decreased Ca^2+^ sensitivity due to downregulation of the BK_Ca_ β1 subunit function (Figs. 2, 3) [210]. BK_Ca_ β1 downregulation during chronic hyperglycemia could be the result of increased protein degradation and/or impaired transcriptional expression of the subunit [210, 211]. Regardless of the mechanism, downregulation of BK_Ca_ β1 expression leads to a reduction in BK_Ca_ channel activity, which is likely to alter vascular smooth muscle function during chronic diabetic hyperglycemia. Intriguingly, no change in BK_Ca_ β1 expression was observed in cultured mouse aortic smooth muscle cells incubated for one week in 25 mM d-glucose compared to control conditions (5.5 mM d-glucose) [51]. The differences are likely due to the use of different vascular beds and culturing conditions. Together, results suggest that short-term chronic exposure of vascular smooth muscle to elevated glucose may alter the functional expression of BK_Ca_ channel subunits. Yet, the physiological implications of these changes remain to be established.

### *K*_*ATP*_* channels*

K_ATP_ channels are octameric protein complexes containing 4 pore-forming K_ir_6.X subunits and 4 accessory sulfonylurea receptor (SUR), likely SUR2B, subunits [14, 212]. These channels are expressed in vascular smooth muscle where they can be regulated by many signaling pathways and molecules, including ATP and ADP [14, 212, 213]. Indeed, whereas elevations in ADP concentration activate the channel, increases in intracellular ATP concentration inhibit K_ATP_ channel function. K_ATP_ channels can also be regulated by ROS signaling and PKC/PKA-mediated pathways [195]. Therefore, K_ATP_ channels can couple vascular smooth muscle metabolic state to electrical activity to regulate cell excitability, myogenic tone and vascular reactivity [14, 212, 213]. Given that hyperglycemia is likely to alter the cellular metabolic state of vascular smooth muscle, it is then reasonable to speculate that K_ATP_ channel activity can be modulated by acute and short-term elevations in extracellular glucose. Surprisingly, however, a study using rat aorta smooth muscle cells found that 24 h elevations in extracellular glucose from 5.5 to 23 mM d-glucose had no effect on K_ATP_ channel activity as measured using single-channel recordings (Table 4) [214]. This study also reported that 23 mM d-glucose impaired the activation of K_ATP_ channels by the anesthetic isoflurane [214], which is known to induce dilation of blood vessels, at least in part, by activating these channels [215, 216]. Although this work did not examine the mechanisms by which elevated glucose impaired the isoflurane effects on K_ATP_ channels, subsequent studies using short-term incubation of human omental artery preparations in elevated glucose suggest that enhanced ROS signaling may be involved [217–220]. Accordingly, incubating endothelium-denuded human omental arteries for at least 60 min in 20 mM d-glucose was sufficient to increase the production of superoxide, likely via a mechanism mediated by activation of PKC and phosphoinositide 3-kinase (PI_3_K)/protein kinase B (Akt) signaling [219, 220]. This excessive superoxide production prevents/reduces endothelium-denuded human omental artery relaxation (as measured using isometric force transducers) in response to application of the K_ATP_ agonist levcromakalim [217–220]. Consistent with these observations, inhibition of PKC and PI_3_K/Akt signaling or treatment with a synthetic peroxisome proliferator-activated receptor γ (PPARγ) agonist that may act as an antioxidant, reduced glucose-induced superoxide production and prevented the impairment in human omental artery relaxation in response to levcromakalim [217–220]. These results suggest, at least indirectly, that elevations in extracellular glucose could impair agonist-mediated activation of K_ATP_ channels in vascular smooth muscle and vasorelaxation. Whether acute and/or short-term elevations in glucose alter basal K_ATP_ channel activity and how this may impact myogenic tone remains unclear. The consequences of glucose-induced alterations in K_ATP_ channel activity are likely to be tissue-specific, as K_ATP_ channels contribute to the regulation of basal myogenic tone in coronary and skeletal muscle arteries but not in other vascular beds [14]. Further studies revealing the effects of acute and short-term glucose exposure on K_ATP_ channel function should attempt to directly measure the activity of K_ATP_ channels and consequences on myogenic tone in different vascular beds.

### Effects of hyperglycemia on sarcoplasmic reticulum ion channels

Vascular smooth muscle cells express a number of sarcoplasmic reticulum (SR) Ca^2+^ release ion channels, including RyR and IP_3_R [14, 105]. These ion channels are instrumental in regulating vascular smooth muscle function.

In vascular smooth muscle, RyRs mediate the localized release of Ca^2+^ from the SR in the form of a Ca^2+^ spark [14, 105, 208]. RyRs in the SR are in close spatial proximity to BK_Ca_ channels located at the surface membrane [14, 105, 208]. This is crucial as RyR-mediated Ca^2+^ sparks are critical for activation of BK_Ca_ channels to produce spontaneous transient outward currents (STOCs), which hyperpolarize vascular smooth muscle leading to relaxation [207, 221]. Thus, changes in the activity of RyRs in response to acute and/or short-term hyperglycemia may have significant consequences on vascular smooth muscle function. Surprisingly, how acute and/or short-term elevations in extracellular glucose impact RyR function, including its coupling to BK_Ca_ channels, in vascular smooth muscle has not been extensively and rigorously examined. A single study has reported that RyR expression may be elevated in cultured A7r5 cells exposed for a minimum of 10 h in 75 mM d-glucose, compared to 25 mM d-glucose [222]. On the other hand, IP_3_Rs mediate localized (e.g. Ca^2+^ puffs) but also cell-wide (e.g. Ca^2+^ waves) Ca^2+^ signals that help regulate vascular smooth muscle function [14, 105, 223, 224]. Like RyRs, changes in IP_3_R function due to acute and/or short-term hyperglycemia may alter vascular smooth muscle function, but studies looking at this are also limited. In a comparative study examining IP_3_R expression in cultured A7r5 cells exposed to 25 or 75 mM d-glucose for at least 10 h, downregulation of IP_3_R protein levels was observed in the higher glucose concentration condition [222]. Conversely, another independent study found no changes in IP_3_R protein expression in cultured A7r5 cells incubated between 3 and 28 days in 5.5 mM and 25 mM d-glucose [225]. The reasons for the disparities between these studies are unclear. Nevertheless, the studies described above for both RyR and IP_3_R highlight major limitations with the use of culturing cells and supraphysiological glucose conditions that may confound interpretation of results. Moreover, given that the mechanisms underlying the changes in RyR and IP_3_R functional expression have not been described, there is a clear opportunity for additional research in this area where physiological conditions are utilized.

### Further considerations

Vascular smooth muscle cells expresses a number of other ion channels [14]. Inward-rectifying K^+^ (K_ir_) channels, various members of the TRP channel family and two members of the T-type Ca^2+^ channel family have all been demonstrated to play essential roles in the regulation of vascular smooth muscle function, myogenic tone and vascular reactivity [14]. Yet, whether the function of these ion channels is altered by acute and short-term elevations in extracellular glucose is unknown. Addressing this issue offers unique opportunities to provide new insights into the regulation of different ion channels, how they are regulated by a highly relevant pathophysiological stimulus and to uncover new mechanisms underlying vascular complications during diabetic hyperglycemia.

Another area that should be considered in future studies relates to mechanisms underlying glucose uptake in vascular smooth muscle. Available studies have shown the expression of several glucose transporters, including insulin-independent Glut1 and insulin-dependent Glut4 in vascular smooth muscle cells [127, 226, 227]. The expression of these glucose transporters can be influenced by glucose concentration both in the intracellular and extracellular milieu. Accordingly, one study found decreased expression of Glut1 upon incubation of cultured rat thoracic aortic smooth muscle in 20 mM d-glucose for 24 h [227]. Interestingly, intracellular glucose levels remained elevated despite the downregulation in Glut1 expression in these cells [227], suggesting that other glucose transporters may also contribute to facilitate glucose uptake in vascular smooth muscle cells. Indeed, in native cerebral vascular smooth muscle cells pre-treated with indinavir, which is considered a selective Glut4 inhibitor [228, 229], elevated glucose-induced potentiation of L-type Ca^2+^ channels was completely blocked [31]. Moreover, Glut4 activity was found to account for 50% of the total glucose uptake in mouse aortic vascular smooth muscle and to be necessary for agonist-induced contraction of mouse thoracic aortic rings [127]. These results suggest a potential role for Glut4 in glucose metabolism in vascular smooth muscle cells. Further studies are necessary to comprehensively understand how glucose enters vascular smooth muscle cells and how this process is regulated.

## Mechanisms impairing vascular ion channels activity during diabetes

Much like in acute and chronic hyperglycemia, vascular smooth muscle function is altered in diabetes, and changes in vascular ion channels expression and/or activity can contribute to this outcome. Below we discuss our current knowledge of the mechanisms altering L-type Ca^2+^ channel, K_V_, BK_Ca_ and K_ATP_ channel activity in vascular smooth muscle during diabetes. RyRs and IP_3_Rs will be briefly discussed given the limited data on the subject.

### Effects of diabetes on L-type Ca^2+^ channels

L-type Ca^2+^ channel activity is distinctly altered in vascular smooth muscle during diabetes (Table 3). Decreased L-type Ca^2+^ channel activity in vascular smooth muscle has been observed in kidney afferent arterioles, aorta and tail arteries from rat STZ models [106–108]. Mechanisms for this reduction in L-type Ca^2+^ channel activity are unclear. Yet, physiological implications of this observation have been suggested to involve impaired responses to vasoconstrictor stimuli that may contribute to altering glomerular filtration, blood flow regulation and even proliferation of vascular smooth muscle [106–108, 230].

Most studies indicate that L-type Ca^2+^ channel activity is increased in vascular smooth muscle from diverse animal models of diabetes [15, 31, 32, 48, 59, 111–115, 231, 232]. Moreover, a recent report showed that the activity of L-type Ca^2+^ channels is also elevated in vascular smooth muscle from diabetic patients [31], suggesting similarities in how diabetes alters the function of this ion channel in animal models and humans. Physiologically, changes in L-type Ca^2+^ channel activity in vascular smooth muscle during diabetes may contribute to altering myogenic tone and vascular reactivity, impairing tissue perfusion and blood pressure, and even triggering the modulation of the contractile and proliferative gene program [14, 15, 51, 230].

The mechanisms underlying the increase in L-type Ca^2+^ channel activity during diabetes are less well understood. However, they do not seem to involve changes in pore-forming subunit expression, as similar protein abundance for Ca_V_1.2 was reported in cerebral/mesenteric and adipose arteries from high fat diet (HFD, type-2 diabetic model) mice and diabetic patients [31, 210]. Rather, current evidence suggests that the effects of diabetes on L-type Ca^2+^ channel function in vascular smooth muscle may be mainly due to post-translational modifications. Accordingly, an initial study examining this issue found that an increase in the expression of the phosphatidylinositol 3-kinase δ isoform (PI3Kδ) underlie the potentiation of L-type Ca^2+^ currents in aortic vascular smooth muscle from a mouse model of type-1 diabetes (STZ) [113]. How PI3Kδ increased L-type Ca^2+^ channel activity during diabetes was not examined. Other studies have suggested that PI3K may act through Akt and protein kinase B (PKB) to increase phosphorylation of the accessory Ca_V_ β subunit that then promotes channel trafficking to the membrane and the stimulation in channel activity [233]. It is thus tempting to speculate that a similar mechanism may be engaged to potentiate L-type Ca^2+^ channel activity in vascular smooth muscle from type-1 diabetic mice. Another study using cerebral vascular smooth muscle from rats in HFD found that increased L-type Ca^2+^ channel activity correlates with elevations in plasma fatty acids, although the specific mechanisms for this were not described [114]. In skeletal artery vascular smooth muscle from STZ rat, increased L-type Ca^2+^ channel activity was associated with enhanced PKC function, but mechanism remains unclear [48].

In dB/dB and HFD models of diabetes, the increase in L-type Ca^2+^ channel activity in cerebral vascular smooth muscle cells was attributed to activation of an unexpected AKAP5-anchored PKA signaling pathway leading to phosphorylation of the Ca_V_1.2 subunit at S1928 (Fig. 2) [31, 112]. Similar results were observed in adipose artery vascular smooth muscle from diabetic patients [31], thus bestowing translational relevance to the aforementioned observations in animal models. Accordingly, genetic depletion of AKAP5, inhibition of PKA, disruption of the interaction between AKAP5 and PKA, and preventing the phosphorylation of the S1928 site halted the increase in L-type Ca^2+^ channel activity in vascular smooth muscle from HFD mice [31]. More recently, the activity of the AC5 isoform was found to be essential for generating local cAMP signaling leading to PKA-dependent potentiation of L-type Ca^2+^ channels in vascular smooth muscle from mice in HFD and STZ [32]. Intriguingly, an independent microarray analysis found that AC5 expression was upregulated in vascular smooth muscle upon chronic hyperglycemic conditions [51]. Augmented AC5 expression may contribute to the formation of additional local complexes between AKAP5, PKA and Ca_V_1.2 that further stimulate L-type Ca^2+^ channel activity during diabetic hyperglycemia. Consistent with this possibility, increased nanometer AC5 and Ca_V_1.2 proximity and localized Ca^2+^ influx frequency (e.g. persistent Ca^2+^ sparklets) were found in vascular smooth muscle from different animal models of diabetes (dB/dB, HFD and STZ) [32, 112]. These results are important as they suggest the engagement of similar signaling pathways in the mechanism altering L-type Ca^2+^ channel activity in different models of diabetes. Moreover, these results are starting to uncover a novel signaling nanocomplex involving AKAP5, AC5, PKA and Ca_V_1.2. This nanocomplex affords a level of compartmentalization that can be selectively engaged to promote cAMP/PKA-dependent phosphorylation of S1928 to stimulate L-type Ca^2+^ channel activity and alter myogenic tone and vascular reactivity during diabetes. Altogether, results suggest altered L-type Ca^2+^ channel activity via multiple mechanisms in vascular smooth muscle that may contribute to modulate myogenic tone and vascular reactivity during diabetes. Results may identify potential new targets (e.g. AKAP5, AC5, S1928) for drug development to treat vascular complications during diabetes.

### Effects of diabetes on K^+^ channels

Most studies report a reduction in K_V_ channel activity in vascular smooth muscle from different vascular beds and animal models of diabetes (Table 4) [199, 200, 204, 234–237]. In coronary vascular smooth muscle from a rat model of type-1 diabetes (STZ), reduced K_V_ function was associated with a decreased expression and increased nitration state of K_V_1.2 but not K_V_1.5 subunits due to enhanced peroxynitrite production [234, 236]. A decrease in Kv7 function was also reported as a potential mechanism altering vascular reactivity in coronary arteries from STZ mice [204]. However, in coronary vascular smooth muscle from HFD mice, decreased K_V_ channel activity was correlated with AGEs-mediated downregulation of K_V_1.2 and K_V_1.5 subunits’ transcript and protein levels [199]. In cerebral and mesenteric vascular smooth muscle from HFD mice, decreased K_V_ channel activity was linked to selective transcriptional suppression of K_V_2.1 subunits via activation of the AKAP5-anchored calcineurin/NFATc3 signaling pathway (Fig. 2) [200]. This change in K_V_2.1 functional expression was correlated with enhanced myogenic tone in HFD cerebral and mesenteric arteries [200]. The studies described above reveal the engagement of distinct mechanisms that could synergize to impair K_V_ channel function and vascular smooth muscle function that may alter myogenic tone and vascular reactivity during diabetes. Future studies should examine whether these mechanisms also alter K_V_ function in vascular smooth muscle from diabetic patients.

BK_Ca_ channel activity is also impaired in vascular smooth muscle cells from different vascular beds, animal models of diabetes, and diabetic patients (Table 4) [42, 210, 211, 238–245]. Indeed, there seems to be consensus among all these studies of a reduction in vascular smooth muscle BK_Ca_ channel activity during diabetes. Moreover, impaired BK_Ca_ channel activity has been associated with impaired vasodilation, increased basal myogenic tone and/or enhanced hyper-reactivity to vasoactive agonists [42, 210, 211, 238–245]. However, in cerebral vascular smooth muscle from dB/dB mice, changes in BK_Ca_ channel activity during diabetes seems to be dependent on biological sex, with females being protected from diabetes-mediated changes in channel function [246]. These results contrast with observations of decreased BK_Ca_ channel activity in adipose artery vascular smooth muscle from both female and male diabetic patients [42], thus suggesting species and/or vessel-specific differences. The general mechanism for reduced BK_Ca_ channel activity has been linked to a downregulation in the functional expression of the BK_Ca_ β1 subunit with no change in expression or function of the pore-forming BK_Ca_ α subunit, or reduced BK_Ca_ β1/ BK_Ca_ α expression ratio [42, 210, 211, 238–245]. Accordingly, reduced BK_Ca_ β1, but not BK_Ca_ α, subunit expression was found in cerebral arteries from a Swiss Webster STZ mouse model [238], aorta from rats and mice in STZ as well as Zucker rat model [211, 239, 241], and aorta, coronary, cerebral, mesenteric arteries from HFD mouse model [210, 245]. In aorta from dB/dB mice, a concomitant increase in BK_Ca_ α subunit and decrease in BK_Ca_ β1 subunit expression was observed [242].

**Fig. 4:**
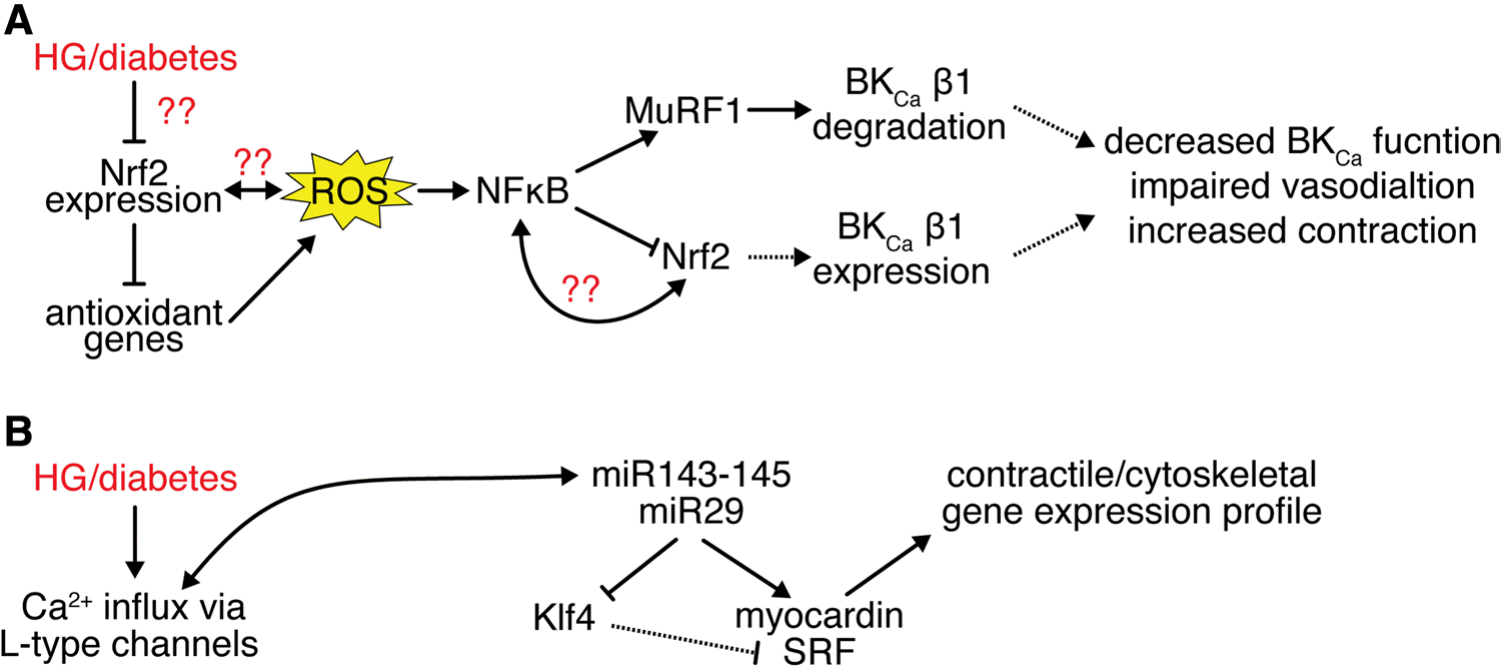
Schematic of models for regulation f gene expression and excitability in vascular smooth muscle during diabetic hyperglycemia. **a** Nrf2 model based on work from Refs. [245, 247]. In this model, diabetic hyperglycemia induces downregulation of Nrf2 via mechanisms that require further examination but that may include NFκB regulation. Decreased expression of Nrf2-regulated genes contributes to ROS production and NF-κB activation. NFκB then inhibits Nrf2-mediated transcriptional induction of BK_Ca_ β1 subunit expression and stimulates MuRF1-mediated degradation of BK_Ca_ β1 protein leading to a reduction in of BK_Ca_ channel activity. Impairment of BK_Ca_ channel function may contribute to altered vascular smooth muscle excitability leading to impaired vasodilation and/or hypercontractility. Key open questions (double question marks) are: (1) how elevations in extracellular glucose and/or diabetes reduce Nrf2 expression, (2) what are the mechanisms regulating the crosstalk between Nrf2 and NFκB? (3) can ROS signaling regulate the functional expression of Nrf2? **b** Proposed model for glucose-induced regulation of vascular smooth muscle contractile and cytoskeletal gene profile based on data from Refs. [51, 263, 265]. Arrows = activation, lines with bars = inhibition, dotted lines = reduced effects.

Several mechanisms have been proposed to account for the reduction in BK_Ca_ β1 subunit expression during diabetes. For instance, NFATc3 activation, via an AKAP5-anchored calcineurin-dependent pathway, was found to mediate downregulation in the expression and function of BK_Ca_ β1 subunit, contributing to enhanced myogenic tone of cerebral arteries in a HFD model of diabetes (Fig. 2) [210]. Other studies reported accelerated BK_Ca_ β1 subunit degradation that is mediated by ROS-dependent activation of a FOXO-3a/FBXO and a NFκB-mediated MuRF1 pathway in mouse aorta and coronary cells (Fig. 4a) [211, 241, 245, 247]. Activation of the NFκB pathway could also lead to suppression of the nuclear factor erythroid-2-related factor 2 (Nrf2) activity and subsequent impairment of Nrf2-mediated transcriptional regulation of the BK_Ca_ β1 subunit expression [245, 247]. Intriguingly, two recent studies reported decreased BK_Ca_ channel activity in vascular smooth muscle from diabetic patients that was mediated by a reduction in the expression and/or function of the BK_Ca_ β1 subunit (Table 4) [42, 243]. One study using adipose artery vascular smooth muscle from diabetic patients showed that impaired BK_Ca_ channel activity was likely due to reduced functional coupling of BK_Ca_ α and β1 subunits rather than changes in the cellular abundance of each subunit or altered Ca^2+^ sparks properties [42]. The other study using coronary artery vascular smooth muscle from diabetic patients reported that altered BK_Ca_ channel function was correlated with downregulation in the protein levels of both BK_Ca_ α and β1 subunits [243]. The differences between these studies and in the mechanisms underlying the alteration in BK_Ca_ channel activity could be due to the use of cells from different vascular beds. Nonetheless, results using human samples are essential to pinpoint similarities and differences with animal models that could help define mechanisms involved in diabetic vasculopathy.

There is substantial evidence suggesting reduced K_ATP_ channel function in vascular smooth muscle from different vascular beds during diabetes that may contribute to impaired cell excitability. Much of this evidence comes from indirect observations showing impaired vasodilation to K_ATP_ openers during diabetes. Accordingly, reduced sensitivity to K_ATP_ openers (e.g. levcromakalim, aprokalin, lemakalim, cromakalim) has been reported in STZ-treated rat aorta [248], cerebral [50], and coronary arteries [249], as well as human coronary arterioles from type-1 and type-2 diabetic patients [36]. Intriguingly, a single study concomitantly examining vascular reactivity in multiple vascular beds in response to the K_ATP_ opener lemakalim found reduced vasodilation in coronary rings, but not in aortic and mesenteric rings from rats in STZ [249], suggesting differential regulation in vascular beds even from the same animal model.

Other studies have correlated the impaired vasodilation to K_ATP_ openers during diabetes to a reduction in K_ATP_ currents (Table 4) [250–252]. In mesenteric vascular smooth muscle from STZ rats, K_ATP_ currents were reported to decrease in a time-dependent manner, reaching a plateau after 14 days of STZ treatment [250]. Although the mechanisms for diabetes-induced alterations in K_ATP_ currents were not examined in this study, it was found that the xanthine derivative KMUP-1 prevented impaired K_ATP_ channel function [250]. Because KMUP-1 can stimulate guanylate cyclase signaling and eNOS activity as well as inhibit phosphodiesterases [253, 254], it is tempting to speculate that this xanthine can simultaneously increase PKB, nitric oxide (NO) and PKA signaling, which converge to improve K_ATP_ channel activity in vascular smooth muscle and rescue vascular function during diabetes. In another study using aorta from STZ rats, decreased K_ATP_ currents in vascular smooth muscle were correlated with a reduction in total protein levels and increase in S-glutathionation of the K_ir_6.X subunit [251]. Enhanced S-glutathionation of K_ir_6.X was suggested to underlie oxidative stress inhibition of K_ATP_ currents [251]. In human umbilical smooth muscle obtained from patients with gestational diabetes, a reduction in K_ATP_ currents was associated with decreased expression of the K_ATP_ channel subunits K_ir_6.X and SUR2B [252]. Interestingly, K_ATP_ currents in diabetic cells were restored to levels observed in control cells by activation of the PKA signaling pathway with forskolin [252]. These results suggest that, in addition to downregulation of K_ATP_ subunits expression, other mechanisms may also contribute to impairing K_ATP_ channel function and K_ATP_-dependent vasodilation during gestational diabetes. Altogether, results suggest that changes in K^+^ channels functional expression due to the engagement of several mechanisms in vascular smooth muscle cells may contribute to the altered myogenic tone and vascular reactivity during diabetes.

### Further considerations

RyR and IP_3_R play a key role in regulating vascular smooth muscle Ca^2+^ dynamics and excitability. However, little is known about how their function is altered in the context of diabetes. A reduction in RyR expression was found in aortic lysates from dB/dB mice [242]. This was linked to a decrease in Ca^2+^ sparks amplitude with no change in frequency in dB/dB cerebral vascular smooth muscle [242]. This study also found that SR Ca^2+^ content was impaired in dB/dB cerebral vascular smooth muscle [242], perhaps indicating modifications in the expression and/or function of the SR Ca^2+^ ATPase SERCA. Indeed, an independent study using rat aorta from the Diabetes Resistant Bio-Breeding (DR-BB) and STZ models of type-1 diabetes found a significant reduction in SERCA2 expression [222]. This alteration may contribute to impair Ca^2+^ spark amplitude in dB/dB cerebral vascular smooth muscle. Ultimately, reduced Ca^2+^ sparks amplitude may alter the RyR-BK_Ca_ channel coupling to impair the negative tonic feedback regulation of contraction mediated by the activation of BK_Ca_ channels. Intriguingly, larger Ca^2+^ spark amplitude was observed in vascular smooth muscle from retinal arterioles of rats in STZ [244]. This is a potential compensatory mechanism to stimulate BK_Ca_ channel activity, which is also impaired due to downregulation of BK_Ca_ β1 in these cells [244]. Whether RyR expression is increased in vascular smooth muscle from STZ-treated rat retinal arterioles is unknown, but a significant elevation in RyR proteins levels has been reported in aorta from STZ rats [222].

The role of IP_3_R in the regulation of vascular smooth muscle excitability during diabetes is less clear. Different reports suggest that intracellular Ca^2+^ transients mediated by IP_3_R are either increased or decreased depending on the species and diabetic model [222, 255]. A decrease and impairment in Ca^2+^ transient dynamics were observed in aortic smooth muscle from DR-BB and STZ rats [222]. These observations were correlated with a decrease in IP_3_R type-1 expression in diabetic cells [222], but the mechanisms were not explored. In stark contrast, an increase in IP_3_-evoked [Ca^2+^]_i_ was found in aortic smooth muscle from dB/dB mice [255]. This effect was directly related to altered stimulation of IP_3_R activity in dB/dB smooth muscle as SR Ca^2+^ content and IP_3_R expression were similar in non-diabetic and diabetic cells [255]. Rather, an increase in the association of IP_3_Rs with the anti-apoptotic protein Bcl-2 was suggested as a potential culprit in enhancing IP_3_-evoked [Ca^2+^]_i_ during diabetes [255], as this protein is known to “sensitize” IP_3_Rs [256]. Indeed, pharmacological inhibition of Bcl-2 restored IP_3_-evoked [Ca^2+^]_i_ transients, but surprisingly did not prevented the agonist-induced hypercontractility in diabetic cells [255]. Thus, the Bcl-2-IP_3_R axis is likely to modulate [Ca^2+^]_i_ to activate other signaling pathways, but play no role in regulation of vascular smooth muscle contraction during diabetes. Taken together, results suggest that diabetes impinges on vascular smooth muscle RyRs and IP_3_Rs via different mechanisms. These data further highlight the need for additional studies examining the functional role of these intracellular ion channels in diabetic vascular complications.

## Vascular smooth muscle, gene expression and diabetic hyperglycemia

Hyperglycemia may also alter vascular smooth muscle excitability by influencing the contractile gene program. For instance, it has been shown that acute elevation in extracellular glucose can stimulate the activation and nuclear accumulation of the transcription factor nuclear factor of activated t cells cytoplasmic 3 isoform (NFATc3) in aorta and cerebral vascular smooth muscle within just 30 min [181, 200]. This time scale for NFATc3 nuclear accumulation seems sufficient to trigger changes in gene expression as altered expression of NFATc3-responsive genes can be observed as soon as 1 h after exposure to elevated glucose [200, 257, 258]. The mechanisms underlying glucose-induced NFATc3 activation seem to involve the stimulation of L-type Ca^2+^ channels to increase Ca^2+^ influx (Fig. 2) [210, 259]. This elevation in [Ca^2+^]_i_ is then “sensed” by calmodulin (CaM), which forms a Ca^2+^/CaM complex that activates the AKAP5-anchored phosphatase calcineurin [134, 210, 260]. AKAP5-anchored calcineurin can then dephosphorylate multiple targets, including NFATc3. Phosphorylated NFATc3 remains inactive in the cytosol, whereas dephosphorylated NFATc3 can translocate into the nucleus where it can alter gene expression, including genes involved in regulation of vascular smooth muscle excitability, such as those coding for K^+^ channels [15, 16, 134]. The reduction of K^+^ channel expression results in membrane potential depolarization, activation of L-type Ca^2+^ channels and alterations in vascular smooth muscle function (see Fig. 2).

Glucose can also regulate the transcriptional activity of Nrf2 in vascular smooth muscle. Nrf2 is a basic leucine zipper transcription factor that plays a significant role in redox homeostasis via modulation of the expression of antioxidant genes [261, 262]. Much like NFATc3, Nrf2 remains in the cytosol where it is found in complex with the E3 ubiquitin ligase, which promotes its degradation in the proteasome to maintain low cellular levels of this transcription factor [261, 262]. In response to changes in cellular oxidative stress, Nrf2 is released from the E3 ubiquitin ligase complex [261, 262]. Free Nrf2 can then translocate to the nucleus where it modulates the expression of genes containing antioxidant responsive elements [261, 262]. Thus, changes in the functional expression of Nrf2 during diabetic hyperglycemia may have profound effects on vascular smooth muscle function, including the regulation of cell excitability. Accordingly, a reduction in Nrf2 protein levels was observed in mesenteric arteriole and aorta lysates from hyperglycemic dB/dB and HFD mice [37, 245, 247], as well as cultured human coronary vascular smooth muscle cells incubated in 22 mM d-glucose (control was cultured cells in 5 mM d-glucose) [247]. In one study, this observation was correlated with downregulation of Nrf2-regulated genes, including many genes coding for antioxidant molecules, and increased production of vascular smooth muscle ROS [37]. Enhanced ROS production in dB/dB cells, perhaps via potentiation of L-type Ca^2+^ channels [124–126], was then suggested to underlie the increased myogenic response in mesenteric arterioles [37]. In a subsequent comprehensive series of experiments using aorta preparations from dB/dB and HFD mice and cultured human coronary vascular smooth muscle exposed to elevated glucose, the decreased Nrf2 expression was correlated with ROS-mediated activation of the transcription factor NFκB and the E3 ubiquitin ligase MuRF1 (Fig. 4a) [245, 247]. Activation of NFκB and MuRF1 during diabetic hyperglycemia were correlated with an Nrf2-dependent reduction in the functional expression of the BK_Ca_ β1 subunit and impaired vasodilation [245, 247]. Intriguingly, these studies show a direct relationship between Nrf2 and BK_Ca_ β1 subunit expression and an inverse relationship between Nrf2 and NFκB/MuRF1 in vascular smooth muscle [245, 247], but the mechanisms for this remains unclear. The schematic in Fig. 4a provides a suggested model integrating observations from the Nrf2 studies in vascular smooth muscle described above during diabetic hyperglycemia.

A recent study used microarray analysis to examine the effects of elevated extracellular glucose, both in vitro and in vivo, on vascular smooth muscle gene expression programming (Fig. 4b) [51]. It was found that chronic glucose stimulation of vascular smooth muscle for as short as 1 week promoted the activation of a contractile and cytoskeletal gene program that could contribute to vascular complications in diabetes [51]. The glucose-induced activation of the contractile/cytoskeletal gene program was mediated by stimulation of myocardin-related transcription factors (MRTF) that translocate to the nucleus to activate serum response factors (SRF) [51]. Repression of the zinc-finger transcription factor Krüppel-like factor 4 (Klf4) was also observed and further hypothesized to contribute to SRF regulation of the contractile gene program [51]. This elegant study found that increased expression of contractile/cytoskeletal markers due to diabetic hyperglycemia was dependent on AGEs, PKC/Rho activation, the expression of the miR143-145 cluster, which can regulate Ca_V_1.2 expression [263, 264], and the activity of L-type Ca^2+^ channels (Fig. 4b) [51]. It was subsequently found that repression of Klf4 expression during chronic hyperglycemia was mediated by miR-29 [265], thus uncovering another layer of regulation. It is intriguing to speculate whether disruption of Ca_V_1.2 S1928 phosphorylation (as in S1928A cells) to prevent potentiation of L-type Ca^2+^ channel activity upon elevated glucose in vascular smooth muscle will normalize the gene expression profile. Nonetheless, glucose-induced activation of contractile/cytoskeletal markers seems to be a general phenomenon of pathophysiological relevance as it was observed in cells from animal models of diabetes and diabetic patients [51]. Overall, these studies highlight mechanisms by which elevated glucose can contribute to regulating vascular smooth muscle function by triggering changes in gene programs. Moreover, this is an exciting area of research that may reveal new targets to treat vascular dysfunction during diabetic hyperglycemia.

### Further considerations

Although outside the scope of this work, hyperglycemia and diabetes may also trigger the activation of a gene program that promotes differentiation of vascular smooth muscle into a synthetic, non-contractile phenotype. The synthetic phenotype supports a proliferative state with altered migratory capacity resulting in the thickening of the intima-media arterial wall [266]. Intriguingly, L-type Ca^2+^ channel activity is essential for activation of both a contractile and synthetic phenotype [51, 230]. The synthetic phenotype has been suggested to contribute to vascular complications, such as atherosclerosis, calcification and inflammation during diabetes [267–271]. However, a role for hyperglycemia in the phenotypic switch is unclear [272]. Nonetheless, activation of both the contractile and synthetic gene program could occur concurrently or via graded activation and/or regulation of different signaling pathways during diabetic hyperglycemia. Accordingly, the synthetic phenotype may be hyperactivated in vascular beds disposed to atherosclerosis, which could also contribute to less responsiveness to vasoactive substances. On the other hand, the contractile phenotype may dominate in blood vessels in which maintaining a level of contraction is essential for proper blood flow and blood pressure regulation (i.e. resistance arteries). Future studies should define whether hyperglycemia itself contributes to the phenotypic switch, especially in an in vivo setting. Moreover, given the "omics era", comparative studies examining how diabetic hyperglycemia regulates contractile and synthetic gene programs in conduit and resistance arteries, are a logical step for future research.

## Therapeutic considerations

The insight gained about the biochemical pathways, signaling generators and ion channels involved in altering vascular smooth muscle function in response to hyperglycemic conditions and diabetes may identify new targets for therapeutic intervention [273]. Indeed, potential treatment options have considered inhibiting (1) the activity of PKC, (2) the generation of AGE and/or activation of RAGE and (3) the generation of ROS [273]. These therapeutic interventions may act by restoring ion channels activity and/or the contractile/synthetic phenotype to basal conditions in vascular smooth muscle (but also in other cell types, such as endothelial cells).

The identification of the P2Y_11_ receptor as a signaling generator promoting glucose-induced PKA-dependent potentiation of L-type Ca^2+^ channels offers an attractive candidate for therapeutic targeting. Inhibition of P2Y_11_ receptors may not only prevent hyperactivity of L-type Ca^2+^ channels during hyperglycemia and diabetes, but also the activation of additional pathological signaling pathways. Indeed, activation of P2Y_11_ receptors have been associated with inflammation, and cardiac and vascular metabolic complications [274], further highlighting the potential for this purinergic receptor as a therapeutic target. Findings indicating that hyperphosphorylation of Ca_V_1.2 at S1928 underlies L-type Ca^2+^ channel potentiation during hyperglycemia and diabetes also open new possibilities to develop improved Ca^2+^ channel blockers that correct impaired channel function (e.g. prevent S1928 hyperphosphorylation), as opposed to established therapies that aim to reduce Ca^2+^ influx. Indeed, despite calcium channel blocker being used to treat cardiovascular complications in diabetic patients [2], clinical trials have shown a modest effect of this drug class on renal and cardiovascular outcomes when compared to other drugs, such as blockers of the renin-angiotensin system [275–277]. Perhaps this new approach of correcting channel function could improve renal and cardiovascular outcome (likely in combination with other drugs, such as renin-angiotensin system inhibitors) [277], and reduce unwanted side effects associated with existing Ca^2+^ channel blockers [278–280].

Targeting K^+^ channels is also an attractive possibility that has been proposed to correct/ameliorate vascular smooth muscle dysfunction in different pathologies, including diabetic hyperglycemia [281]. For example, treatment options that increase functional expression of BK_Ca_ β1 subunit may contribute to rescue vascular smooth muscle function. Accordingly, the use of dimethyl fumarate (DMF), a Food and Drug Administration (FDA) approved Nrf2 activator, has been shown to rescue BK_Ca_ β1 subunit, BK_Ca_ channel activity and sheer-stress induced coronary vasodilation in dB/dB and HFD mice [245, 247]. Inhibition of NFATc3 has also been shown to prevent BK_Ca_ β1 and K_V_2.1 subunits downregulation in vascular smooth muscle and rescue vascular function in HFD mice [200, 210]. It is also intriguing to speculate that AGE/RAGE inhibitors could contribute to improve vascular smooth muscle function by preventing or restoring K_V_ subunit expression [199]. The use of K^+^ channel openers is also a therapeutic option that has been proposed for many years due to their antihypertensive properties [282, 283]. However, as discussed above, the use of a given K_ATP_ opener itself does not rescue vascular reactivity, at least in some vascular beds. The use of compounds, such as KMUP-1 [250], may rescue other signals (e.g. NO, PKA) to restore K_ATP_ activity and vascular function. Consistent with this, perhaps a pharmacological approach targeting many of these ion channels, signaling generators and signaling pathways will be essential for the long-term success in the treatment of vascular smooth muscle dysfunction and vascular complications during diabetic hyperglycemia.

## Conclusion

Diabetes is a multifactorial disorder and a major risk factor for cardiovascular diseases. The vasculature is particularly sensitive to the detrimental effects of diabetes. Hyperglycemia plays a central role in mechanisms underlying both vascular endothelial and smooth muscle dysfunction. Whereas the molecular and cellular mechanisms underlying endothelial dysfunction during hyperglycemia and diabetes have been extensively studied [3, 7, 21–24], much less is known about how vascular smooth muscle is affected. Here we chose to focus on how vascular smooth muscle ion channels are affected by hyperglycemia and diabetes. We did so because ion channels are (1) essential for the regulation of vascular smooth muscle function, and (2) heavily impacted by hyperglycemia and diabetes. In our narrative, we deemed relevant to briefly discuss the information available on how hyperglycemia and diabetes may influence the functional expression of ion channels in endothelial cells as well as the underlying consequences in myogenic tone and vascular reactivity. We then dove into examining the growing body of literature describing how glucose and diabetes can promote changes in the activity of ion channels (particularly L-type Ca^2+^ channels, K_V_/BK_Ca_/K_ATP_ K^+^ channels, RyRs and IP_3_R) in vascular smooth muscle. We summarized mechanisms underlying changes in their function, when possible attempted to reconcile differences, integrate observations and identify knowledge gaps, described how these changes could be related to alterations in myogenic tone and vascular reactivity, and highlighted potential therapeutic considerations. A key take-home message is how one can leverage glucose as a stimulatory molecule to uncover and characterize novel signaling pathways that play a central role in controlling vascular smooth muscle excitability (and perhaps the phenotypic profile). It is clear that mechanisms underlying ion channels dysfunction during hyperglycemia and diabetes are variable, but not necessarily mutually exclusive. It is also clear that vascular smooth muscle ion channels function in response to hyperglycemia and diabetes can be distinctively modulated depending on species, vascular bed and general experimental conditions, such as hyperglycemic and diabetic state. Moreover, the single mechanism approach that is often employed taking advantage of in vitro tools, has to be corroborated in vivo to account for a complex integration of many factors at the organism level. Indeed, implementation of computational modeling could provide an advantage in this regard by allowing the integration of multiple factors that interact non-linearly to affect outcomes and inform experimental designs to be tested in vivo. As our knowledge of the functional expression of other ion channels in vascular smooth muscle expands, it is likely that those newly discovered channels also contribute to vascular smooth muscle dysfunction, thus opening additional research and therapeutic opportunities.
